# DDD-costs have a strong influence on antibacterial drug prescription in Germany: a differentiated correlation analysis from 1985 to 2022

**DOI:** 10.1007/s00210-024-03288-0

**Published:** 2024-07-23

**Authors:** Lilly Josephine Bindel, Roland Seifert

**Affiliations:** Institute of Pharmacology, Hannover Medical School, 30625 Hannover, Germany

**Keywords:** Antimicrobial consumption, Antibiotic, Antimicrobial prescription, Antibiotic prescription, Germany, Arzneiverordnungsreport, Surveillance, Antibiotic stewardship, Correlation, Socioeconomic factors

## Abstract

Antibacterial drugs are vital in modern medicine, and understanding the factors influencing their prescriptions is essential for maintaining their effectiveness and accessibility. This study investigates the hypothesis that costs significantly impact the prescriptions of antibacterial drugs, a factor previously underrecognized. To investigate this, we conducted correlation analyses on defined daily dose (DDD-) prescriptions and DDD-costs for the 15 most prescribed antibacterial drugs in Germany in 2022, using data from the Arzneimittelverordnungsreport (Drug Prescription Report) (1985–2022). The analysis focused on the periods 1985–2022, 1985–2011, and 2012–2022. Our findings revealed significant correlations between DDD-prescriptions and -costs for nearly all drugs over the entire period (1985–2022), with strong negative correlations for amoxicillin (− 0.941), cefuroxime axetil (− 0.900), clindamycin (− 0.800), nitrofurantoin (− 0.895), and cefaclor (− 0.819). From 1985 to 2011, only significant negative correlations were observed. In 2012–2022, significant correlations included both negative and positive, with strong positive correlations for ciprofloxacin (0.950) and clarithromycin (0.962). Overall, DDD-costs showed a persistent and strong correlation with DDD-prescriptions, particularly in the earlier period. This influence appears to diminish slightly in recent years, suggesting other factors might become more significant as DDD-costs plateau. Notably, amoxicillin, amoxicillin clavulanic acid, and nitrofurantoin consistently exhibited strong cost-prescription correlations throughout the entire period. While correlation does not imply causation, the significant and strong correlations, coupled with logical explanations, support the hypothesis that costs are a primary factor in prescribing patterns. Further research is needed to establish causality and examine other potential systemic factors affecting antibacterial drug prescriptions.

**Supplementary Information:**

The online version contains supplementary material available at 10.1007/s00210-024-03288-0.

## Introduction

Antibacterial drugs are essential in modern healthcare for combating infectious diseases. However, their effectiveness is increasingly threatened by bacterial resistance, exacerbated by indiscriminate use (BMG 2023). Additionally, supply chain disruptions, particularly for amoxicillin, amoxicillin clavulanic acid, and penicillin, pose significant challenges to their availability (BfArM 2023).

To ensure the effectiveness and accessibility of antibacterial drugs, it is important to understand the underlying systemic influencing factors for individual prescription development. Previous research has primarily focused on individual-level prescription behavior of physicians and patients (Acampora et al. 2024; Queder et al. 2022), and on healthcare-related factors such as guidelines and financial investments (Kasse et al. 2024). Consequently, suggested improvements have mainly regarded the knowledge and communication in primary healthcare settings (Magin et al. 2022).

Despite this, prior research by our group suggests that costs may exert a greater influence on prescribing practices than previously considered (Bindel and Seifert 2024). To prove this hypothesis, we performed correlation analyses to quantify this influence. If confirmed, a focus on systemic-wide measures might be more effective than the current individual-level approach.

Our analysis evaluated the relationship between DDD-costs and DDD-prescriptions from 1985 to 2022. We also divided this period at 2011 to examine changes over the last decade. Focusing on the 15 most prescribed antibacterial drugs in Germany, based on the 2023 Arzneiverordnungsreport, we aim to understand patterns in prescription behavior. This understanding will enable proactive measures and facilitate assessing the impact of DDD-costs on prescription behavior.

## Materials and methods

### Data collection

The analysis of DDD-prescriptions and DDD-costs of antibacterial drugs is based on the Arzneiverordnungsreport (AVR, Drug prescription report) for the years 1985 to 2022 (Schwabe and Paffrath 1985, 1986, 1987, 1988, 1991, 1992, 1993, 1994, 1995, 1996, 1998, 1999, 2000, 2001, 2002, 2003, 2004, 2006, 2007, 2008, 2010a, b, 2011a, b, 2012, 2013, 2014, 2015, 2016, 2017; Schwabe et al. 1989, 1990, 2018, 2019; Schwabe 1997; Schwabe and Ludwig 2020; Ludwig et al. 2021, 2023, 2024). Since we focused on outpatient prescriptions, only the general chapter “Antibiotika und Chemotherapeutika” (antibiotics and chemotherapeutics) was considered. Therefore, specialized subchapters like urology, dermatology, and ophthalmology were excluded.

### Selection of drugs

For a general overview, we collected data from categorized substance classes. We only included antibacterial drugs, resulting in the exclusion of groups like antimycotics, antiretroviral drugs, and antivirals. Particularly interesting were the most prescribed drugs, for which we selected the TOP15 based on the year 2022.

### Preparation of data

**Fig. 1:**
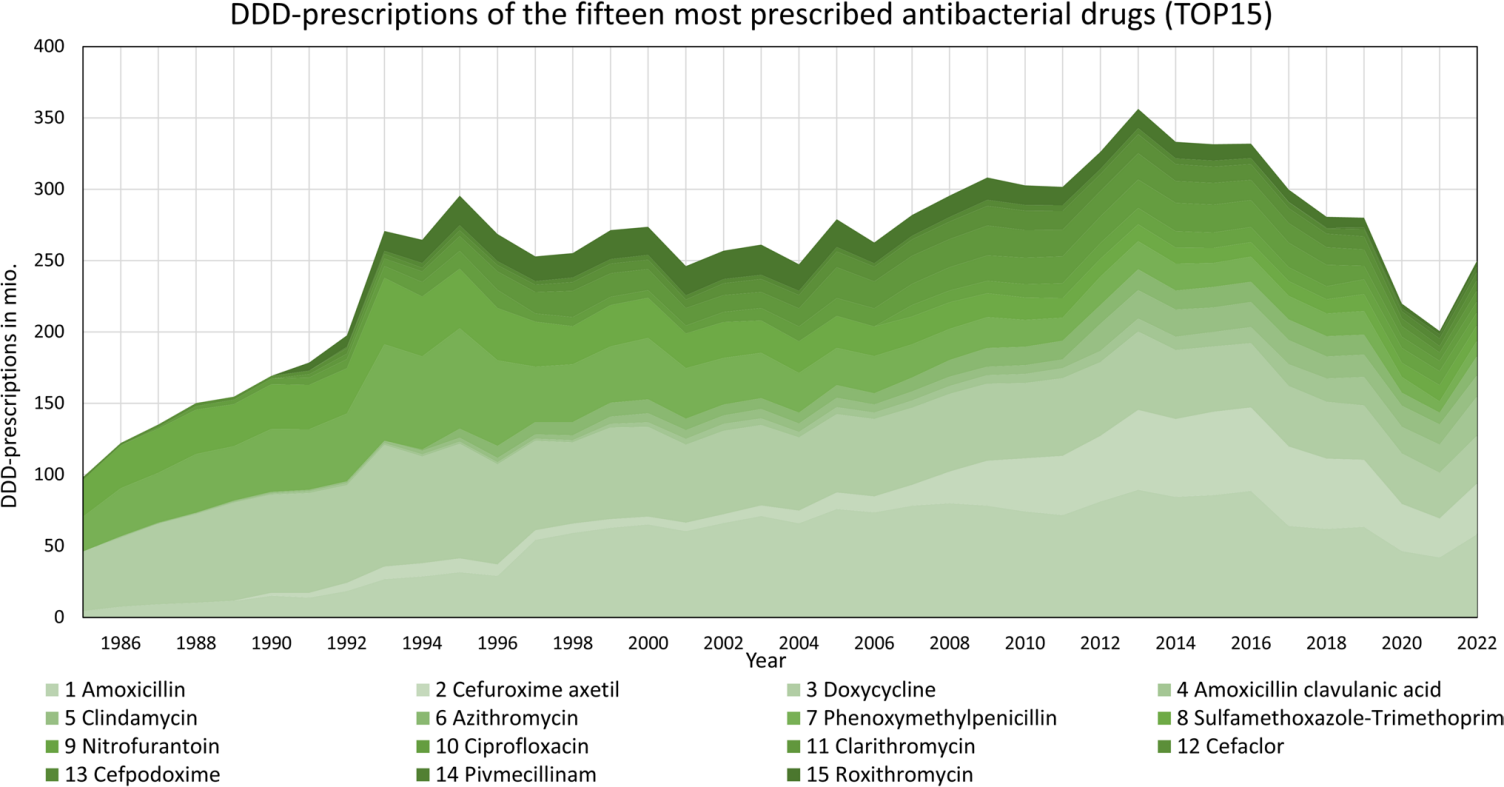
Development of the DDD-prescriptions for the 15 most prescribed antibacterial drugs in 2022 (TOP15). The absolute values are shown as a stacked area

**Fig. 2:**
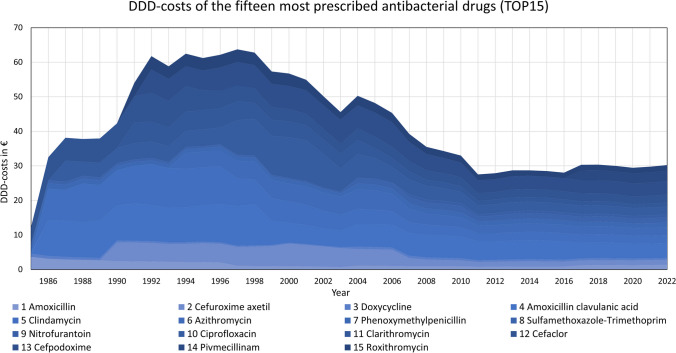
Development of the DDD-costs for the 15 most prescribed antibacterial drugs in 2022 (TOP15). The absolute values are shown as a stacked area

After collecting data for DDD-prescriptions (Fig. 1) and DDD-costs (Fig. 2), in some cases, processing initial data was necessary. This included currency conversion from the German Mark to the Euro and calculating proportionate mean costs when several preparations were depicted.

SPSS was used to analyze the correlations between DDD-prescriptions and DDD-costs. A bivariate correlation analysis was performed using the Pearson correlation coefficient. The coefficient of determination (R^2^) was calculated manually. Results were processed and visualized using SPSS and Excel.

### Analysis of the correlations and presentation of data

To provide a deeper insight into the relationship between DDD-costs and DDD-prescriptions, we conducted three different analyses. The first correlation analysis covered the entire period from 1985 to 2022. To determine whether the relationship changed over time, we divided the time span into two further periods, setting the point of separation at 2011. This division allowed us to examine the development of the last 10 years separately, considering that since 2011, DDD-costs have approached a plateau, suggesting a potential trend reversal. This is depicted in Fig. 2.

During the analysis, we emphasized three aspects of the correlations: significance, direction (positive vs. negative), and strength. The Pearson coefficient indicates whether there is a linear relationship between the two variables. The correlation coefficient ranges from − 1 to + 1. A positive coefficient indicates that both variables influence each other in the same direction, while a negative coefficient indicates an inverse relationship. A value of 0 signifies no linear relationship, while a value of 1 indicates a very strong linear relationship with same proportions of growth (Mukaka 2012). We define a correlation above ( ±) 0.8 as strong, indicating a substantial influence between both factors. Values below 0.8 suggest a weaker relationship.

Beside the correlation coefficient, the significance of the correlation should be considered. The significance level indicates the extent to which the values can be generalized and considered reliable. Only significant values validate the correlation coefficient, allowing to draw conclusions. If there is non-significance, the values are only limited informative. A value with a significance level of 0.01 as well as 0.05 is determined to be considered significant.

The coefficient of determination (R^2^), calculated by squaring the Pearson correlation coefficient, indicates the proportion of the variance in the dependent variable that is predictable from the independent variable. Beside the significance, it can be used as an indicator whether the given correlation is valid.

Figures 1, 2, and 3 and Tables 1 and 2 show the most important results of our study. Additional information is found in the appendix in supplemental Figures S1–S15 and Tables S1–S43.

**Fig. 3:**
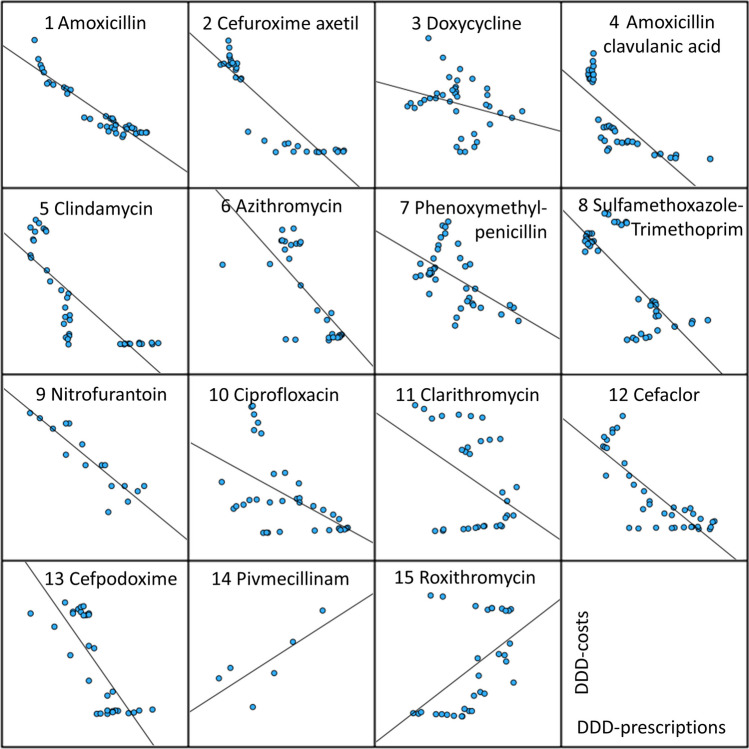
Correlation between DDD-prescriptions and DDD-costs for the 15 most prescribed antibacterial drugs (TOP15) in 2022 (Ludwig et al. 2024). Depicted are the correlation panels for the entire period (1985–2022). The DDD-prescriptions are plotted on the X-axis and the DDD-costs on the Y-axis

**Table 1:**
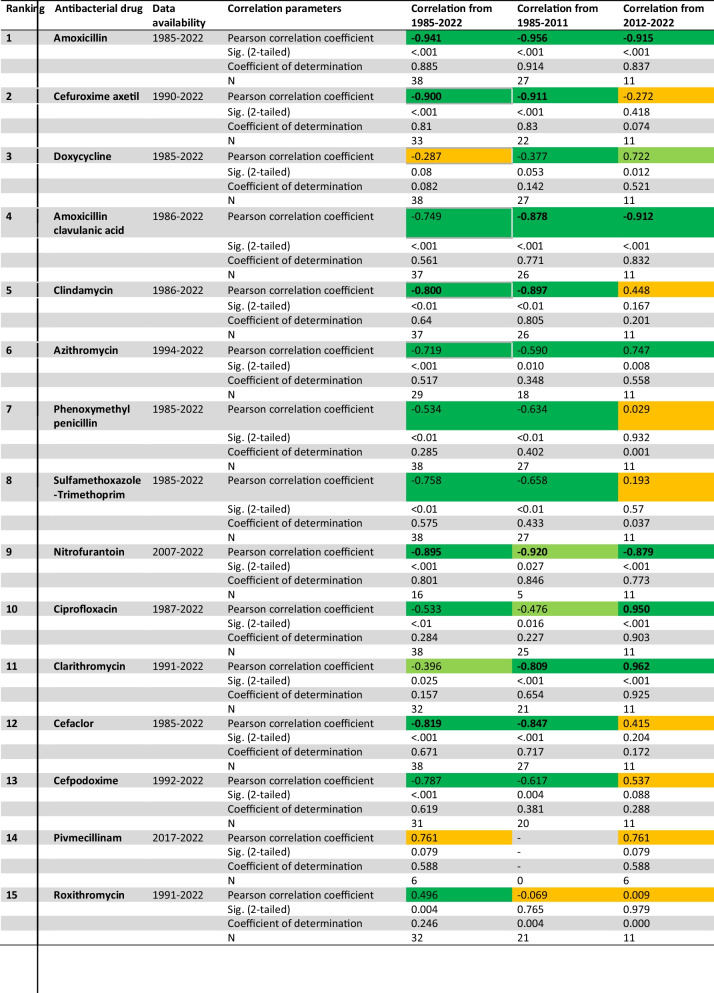
Correlation between prescriptions defined daily dose and DDD-costs for the 15th most prescribed antibacterial drugs (TOP15) in 2022 (Ludwig et al. 2024). Three different correlations were performed, which differ in the time interval under consideration. The entire period from 1985 to 2022 was analyzed, as well as an earlier section from 1985 to 2011 and a later one from 2012 to 2022. Dark green color indicates a significant correlation at the 0.01 level. Light green color indicates a significant correlation at the 0.05 level. Orange color indicates no significant correlation

**Table 2:** Summary of aspects regarding the correlations of DDD-prescriptions vs. DDD-costs

	Correlation from 1985–2022	Correlation from 1985–2011	Correlation from 2012–2022
Average number of analyzed years (max.; min.)	30 (38; 6)	22.79 (27; 0)	10.67 (11; 6)
Number of total correlations	15	14	15
Number of significant correlations	13	13	7
Number of strong correlations	5	7	5
Number of non-significant correlations	2	1	8
Number of significant positive correlations (strong)	1 (0)	0 (0)	4 (2)
Number of significant negative correlations (strong)	12 (5)	13 (7)	4 (3)
Share of significant vs. total correlations	86.66%	92.86%	46.66%
Share of strong vs. total correlations	33.33%	50.00%	33.33%

## Results

### Correlation of prescriptions vs. costs during the entire available period

An analysis of the correlation for DDD-prescriptions vs DDD-costs was conducted for the 15 most prescribed antibacterial drugs (TOP15) in 2022 (Ludwig et al. 2024). In these analyzed drugs, various substance groups are represented. Amoxicillin and amoxicillin clavulanic acid belong in the group of aminopenicillins. Cefuroxime axetil, cefaclor, and cefpodoxime are cephalosporins. Azithromycin, clarithromycin, and roxithromycin are macrolides, while clindamycin is a lincosamide. Doxycycline belongs in the group of tetracyclines and nitrofurantoin belongs to the group of other anti-infective chemotherapeutics. Phenoxymethylpenicillin and pivmecillinam are penicillins and ciprofloxacin belongs in the group of fluoroquinolones.

The data cover a period from 1985 to 2022, as AVRs have been published for these years. However, data are not available for all preparations from 1985 onwards, since some antibacterial drugs were not yet launched on the market or were not included in the TOP3000 drugs listed in the AVR. Data are available from 1985 for amoxicillin, doxycycline, phenoxymethylpenicillin, sulfamethoxazole-trimethoprim, and cefaclor. A few years later, data became available for amoxicillin clavulanic acid (1986), clindamycin (1986), ciprofloxacin (1987), cefuroxime axetil (1990), clarithromycin (1991), roxithromycin (1991), and cefpodoxime (1992). Initial data for azithromycin (1994), nitrofurantoin (2007), and pivmecillinam (2017) were published comparatively late. All drugs have been listed consecutively since their first recording. The data availability for each drug for the analyzed years can be found in Table 1.

Significant correlations were observed for nearly all drugs except doxycycline and pivmecillinam. Clarithromycin has a significant correlation at the 0.05 level, while all other antibacterial drugs have correlation coefficients at the 0.01 level. All correlation parameters can be found in Table 1 and in S1–S43, as well as a graphical illustration in Fig. 3.

A significant negative correlation coefficient was found for amoxicillin (− 0.941), cefuroxime axetil (− 0.900), amoxicillin clavulanic acid (− 0.749), clindamycin (− 0.800), azithromycin (− 0.719), phenoxymethylpenicillin (− 0.534), sulfamethoxazole-trimethoprim (− 0.758), nitrofurantoin (− 0.895), clarithromycin (− 0.396), cefaclor (− 0.819), and cefpodoxime (− 0.878). A non-significant correlation coefficient was observed for doxycycline (− 0.287).

A significant positive correlation is only depicted for roxithromycin (0.496). Although there is a positive correlation for pivmecillinam (0.761), it is not considered since it is not significant.

Strong correlations, defined as a correlation coefficient above ( ±) 0.8, were also identified. All strong correlation coefficients are negative correlation coefficients of DDD-prescriptions and DDD-costs. They were observed for amoxicillin (− 0.941), cefuroxime axetil (− 0.900), clindamycin (− 0.800), nitrofurantoin (− 0.895), and cefaclor (− 0.819).

### Correlation of prescriptions vs. costs until 2011

To provide a detailed and differentiated insight into the correlations, the period from the beginning of the records up to and including 2011 was analyzed. For some antibacterial drugs, data is available for the entire period from 1985 to 2022, while for others, data is only available from a later period. These were analyzed from the period of their first listing up to 2011. There is no correlation for pivmecillinam, as data was only published from 2017 onwards.

Significant correlations are available for all antibacterial drugs except roxithromycin with a non-significant correlation and pivmecillinam due to a lack of data. Nitrofurantoin and ciprofloxacin have significant correlation coefficients at the 0.05 level, while all other drugs have a correlation coefficient at the 0.01 level. All correlation parameters can be found in Table 1 and S1–S43.

No positive correlations were observed. All 14 analyzed drugs exhibit negative correlations, with thirteen being significant and one non-significant correlation for roxithromycin (− 0.069).

Strong negative correlations, defined as a correlation coefficient above ( ±) 0.8, are available for amoxicillin (− 0.956), cefuroxime axetil (− 0.911), amoxicillin clavulanic acid (− 0.878), clindamycin (− 0.897), nitrofurantoin (− 0.920), clarithromycin (− 0.809), and cefaclor (− 0.847).

Additionally, significant negative correlations that are not classified as strong were observed for doxycycline (− 0.377), azithromycin (− 0.590), phenoxymethylpenicillin (− 0.634), sulfamethoxazole-trimethoprim (− 0.658), and cefpodoxime (− 0.617).

### Correlation of prescriptions vs. costs from 2012 to 2022

To compare an earlier period with developments over the last 10 years, the correlation of DDD-prescriptions and DDD-costs was analyzed from 2012 to 2022. Data were available for almost all antibacterial drugs for this period, except for pivmecillinam, for which data are only available from 2017 onwards, resulting in fewer years being analyzed. All correlation parameters can be found in Table 1 and S1–S43.

For several drugs, the correlation is not significant. These include cefuroxime axetil (− 0.272), clindamycin (0.448), phenoxymethylpenicillin (0.029), sulfamethoxazole-trimethoprim (0.193), cefaclor (0.415), cefpodoxime (0.537), pivmecillinam (0.761), and roxithromycin (0.009).

Significant positive and negative correlations were observed for all other drugs. A significant negative correlation was found for amoxicillin (− 0.915), amoxicillin clavulanic acid (− 0.912), and nitrofurantoin (− 0.879). Significant positive correlations were shown for doxycycline (0.722), azithromycin (0.747), ciprofloxacin (0.950), and clarithromycin (0.962).

Strong correlations, defined as a correlation coefficient above ( ±) 0.8, were both positive and negative. While all significant negative correlations were strong, this was not the case for the positive correlations. Strong positive correlations were observed for ciprofloxacin (0.950) and clarithromycin (0.962).

## Discussion

### Development of DDD-prescriptions

Prescription trends for various drugs exhibit recognizable patterns. Certain exceptions exist, often due to strong distortions, like those seen in clindamycin. All prescription curves for the TOP15 are illustrated in Fig. 1 and S1–S15. A decreasing trend in recent years is shown for amoxicillin, cefuroxime axetil, clindamycin, cefaclor, phenoxymethylpenicillin, sulfamethoxazole-trimethoprim, and doxycycline (see Figs. S1, S2, S5, S7, S8, S10, and S12). Amoxicillin and cefaclor became popular in the mid-1990s and showed a strong increase in prescriptions. Around 2010, both showed a plateau until a decline set in, with cefaclor around 2014 and amoxicillin around 2017. Cefuroxime axetil and clindamycin (see Figs. S2 and S5) showed slow growth at a very low level at the beginning, followed by a strong increase, for cefuroxime axetil from 2007 and for clindamycin a jump in prescriptions in 2012. In recent years, after a plateau, a decline set in for both. In clindamycin and phenoxymethylpenicillin (see Figs. S5 and S7), a strong distortion occurred due to the inclusion of dental prescriptions in 2012 (Schwabe and Paffrath 2013). Doxycycline, phenoxymethylpenicillin, and sulfamethoxazole-trimethoprim (see Figs. S3, S7, and S8) became popular in the 1990s. At this time, all three of them showed a quick increase, followed by an ongoing decrease. A similar case exists with ciprofloxacin and cefaclor (see Figs. S10 and S12). Both of them increased in 2002 and started decreasing in 2014.

Antibacterial drugs showing an increasing trend are amoxicillin clavulanic acid, cefpodoxime, and pivmecillinam (see Figs. S4, S13, and S14). Amoxicillin clavulanic acid has shown an increase in prescriptions that is gathering pace since the beginning. Cefpodoxime shows an increasing trend too, with a more rapid increase since 2019. Pivmecillinam is a relatively new drug with a market launch in 2016. Since then, it has shown steadily growing prescription rates. All three drugs show a stringent increasing trend without strong declines.

### Development of DDD-costs

Like the trends observed in prescriptions, the correlations in costs also reveal distinct patterns. In most cases, decreasing costs are evident. However, rising DDD-costs are occurring in nitrofurantoin and pivmecillinam. All curves of DDD-costs for the TOP15 are illustrated in Fig. 2 and S1–S15.

Several characteristic patterns emerge in the cost curves. A sudden, sharp drop in prices is typically seen when the initial price reduction is remarkable. A continuous, long-term decline suggests sustained cost reductions over time. Drugs like cefuroxime axetil, azithromycin, and cefpodoxime show a sudden drop followed by a plateau (see Figs. S2, S6, and S13). On the other hand, doxycycline, clindamycin, phenoxymethylpenicillin, sulfamethoxazole-trimethoprim, clarithromycin, and cefaclor exhibit a more gradual, continuous price reduction without a sudden jump at the beginning (see Figs. S3, S5, S7, S8, S10, and S12). A combination of these characteristics is observed in amoxicillin, amoxicillin clavulanic acid, ciprofloxacin, and roxithromycin (see Figs. S1, S4, S10, and S15).

In some cases, cost increases are observed before sharp decreases, notably in ciprofloxacin and cefaclor (see Figs. S10 and S12). Smaller increases occur in cefuroxime axetil, amoxicillin clavulanic acid, clindamycin, and azithromycin (see Figs. S2, S4, S5, and S6). A sudden spike in costs around 2004 is noted across many drugs, likely due to the pharmaceutical law GMG 2004 (Schwabe and Paffrath 2007). Doxycycline, phenoxymethylpenicillin, sulfamethoxazole-trimethoprim, and roxithromycin show particularly high increases, suggesting distortion of the cost curve (see Figs. S3, S7, S8, and S15).

A plateau formed in most cases around 2011, suggesting that strong cost reductions may have ceased due to several factors. In addition to the AMNOG law in 2011 (AOK 2023), supply shortages, delivery bottlenecks, or a profitability limit reached by the pharmaceutical industry could explain this stabilization.

Overall, most drugs show a decrease in costs, with some notable fluctuations. Rising DDD-costs are observed in sulfamethoxazole-trimethoprim and pivmecillinam (see Figs. S7 and S14). While pivmecillinam is still patent-protected, sulfamethoxazole-trimethoprim’s costs increased so much in 2004 that they remain higher today than at the start of the recordings. Though many drugs show a consistent downward trend, others exhibit more pronounced fluctuations.

Specific events, such as changes in pharmaceutical laws or market dynamics, can lead to significant shifts in cost trends. The notable changes in 2004 and 2011 are likely due to GMG 2004 and AMNOG 2011, respectively. Although the COVID-19 pandemic had profound effects on prescriptions, it does not appear to have impacted DDD-costs in the TOP15 drugs (see Fig. 2).

### Correlation of prescriptions vs. costs during the entire available period

During the entire period from 1985 to 2022, a significant and strong negative correlation exists between prescriptions and costs for most analyzed antibacterial drugs (see Table 1 and Fig. 3). Exceptions include doxycycline and pivmecillinam. The non-significance in doxycycline can be attributed to a sudden increase in costs in 2004 due to the measures of the drug law GMG, which introduced a standardized pharmacy dispensing fee (Schwabe and Paffrath 2007). Pivmecillinam, being a relatively new drug, has not yet experienced patent expiry, and thus, no significant cost reduction has been realized due to the lack of generics (see Fig. S14).

The negative correlation indicates that decreasing costs are associated with rising prescriptions for almost all drugs, except roxithromycin and pivmecillinam (see Table 1, S14 and S15). The positive correlation for roxithromycin can be explained by its long-term downward trend in prescriptions, where falling costs did not reverse this trend (see Fig. S15).

Drugs with a correlation coefficient exceeding ( ±) 0.8, indicating a strong correlation, include amoxicillin (− 0.941), cefuroxime axetil (− 0.900), clindamycin (− 0.800), nitrofurantoin (− 0.895), and cefaclor (− 0.819). These drugs typically exhibit a sharp increase in prescriptions following significant price reductions (see Figs. 1 and 2; Figs. S1, S2, S5, S9, and S12). This pattern underscores the importance of DDD-costs as a key factor influencing prescriptions, particularly for the most frequently used antibacterial drugs.

### Correlation of prescriptions vs. costs until 2011

In the analysis of the early period of data recording, only negative correlations are depicted. Except the non-significance of roxithromycin and the lack of data for pivmecillinam, all other antibacterial drugs show a significant value (see Table 1). The non-significance of roxithromycin might be explainable through a sudden increase in costs in 2004, as shown in Fig. S15. This strong uplift is caused by the measures of the drug law GMG, due to a standardized pharmacy dispensing fee (Schwabe and Paffrath 2007). Beside this, DDD-prescriptions were rising before a decline in DDD-costs took place. This might indicate that other factors were more important than DDD-costs for roxithromycin in the time period of 1991–2011.

There are no positive correlations depicted, supporting the suggestion of declining costs. Since a quite long time span of 27 years is considered, most of the depicted antibacterial drugs experienced the introduction of generics, followed by declining costs. Since the most popular drugs from today’s perspective are analyzed, it can be assumed that they increased in popularity at least during a certain period of time in order to reach their current level.

Beside the fact that only negative correlations are available, many of them are considered strong as they have a correlation coefficient of over ( ±) 0.8. The TOP5 have a very high proportion of strong on significant correlations again. In the TOP5, four of five drugs show a strong negative correlations, while among the TOP6-15, three of nine drugs have a correlation considered strong.

### Correlation of prescriptions vs. costs from 2012 to 2022

In the analysis of the late period of data recording, both positive and negative correlations are depicted. Strong correlations can be both positive and negative; noticeable is the high number of non-significant correlations (see Table 1).

Non-significant correlations are depicted for eight drugs: cefuroxime axetil (− 0.272), clindamycin (0.448), phenoxymethylpenicillin (0.029), sulfamethoxazole-trimethoprim (0.193), cefaclor (0.415), cefpodoxime (0.537), pivmecillinam (0.761), and roxithromycin (0.009). This large number can be explained by the comparable short time period of 11 years under consideration combined with a strong effect of the COVID pandemic in 2020 and 2021 on prescriptions (see Fig. 1). Due to the small number of data analyzed, outliers are particularly influential.

Regarding the significant correlations, four of them are positive and three of them are negative. The share of strong correlations is balanced too, with two negative and three strong positive correlations. A summary of aspect characterizing the analyses can be found in Table 2.

Positive correlations are depicted for doxycycline (0.722), azithromycin (0.747), ciprofloxacin (0.950), and clarithromycin (0.962). These drugs show a decreasing trend in DDD-prescriptions and slightly decreasing DDD-costs (see Figs. S3, S6, S10, and S11). Negative correlations are shown for amoxicillin (− 0.915), amoxicillin clavulanic acid (− 0.912), and nitrofurantoin (− 0.879). While decreasing DDD-prescriptions and increasing DDD-costs are occurring in amoxicillin and nitrofurantoin (see Figs. S1 and S9), amoxicillin clavulanic acid shows increasing DDD-prescriptions and decreasing DDD-costs (see Fig. S4).

A suggestion for the comparable large number of positive correlations might be that other factors are becoming more important than DDD-costs on prescription figures. This is supported by the DDD-costs reaching a plateau in most drugs in recent years (see Fig. 2).

The TOP5 are outstanding by their comparatively high number of significant as well as strong correlations. Regarding the number of significant correlations, the TOP5 have three of five correlations which are significant vs. the TOP6-15 with four of ten. While two of the five strong correlations are presented in the TOP5, only three belong to the TOP6-15 (see Table 1).

### Comparison of the entire period and time segments

The correlations between DDD-prescriptions and DDD-costs were analyzed for three distinct time spans: the entire period from 1985 to 2022, the early period from 1985 to 2011, and the recent period from 2012 to 2022. This comparison aims to identify similarities and differences in these correlations across the different intervals. A summary of aspects regarding the three analyses can be found in Table 2, while all correlation parameters are depicted in Table 1 and S1–S43.

The changes in correlation coefficients of individual antibacterial drugs can be categorized into three main patterns. Some drugs, such as amoxicillin, amoxicillin clavulanic acid, and nitrofurantoin, exhibit consistent negative correlations across all periods. The strength of these correlations remains similar and significant, indicating a stable relationship where decreasing costs are associated with increasing prescriptions. In contrast, drugs like cefuroxime axetil, clindamycin, phenoxymethylpenicillin, sulfamethoxazole-trimethoprim, cefaclor, and cefpodoxime show significant negative correlations in the early period and the entire period. However, in the recent period, the correlation becomes non-significant or changes direction from negative to positive. This suggests a weakening influence of DDD-costs on DDD-prescriptions and the potential emergence of other influencing factors. Lastly, it is important to note that the data for pivmecillinam is insufficient for robust analysis as it has only been available since 2017.

The consistency of the relationship between DDD-prescriptions and DDD-costs for drugs like amoxicillin, amoxicillin clavulanic acid, and nitrofurantoin suggests that their cost dynamics have remained relatively stable over time. Conversely, the changes observed for drugs like cefuroxime axetil and clindamycin indicate that factors other than cost might be influencing prescription trends in the recent period. The observed shift from significant to non-significant correlations, or from negative to positive correlations, in the recent period may be attributed to a variety of factors. These include the smaller number of data points available for analysis, which affects the significance of the results, and the potential impact of external influences such as the COVID pandemic on prescription patterns.

Overall, the analysis of the entire period reveals a higher proportion of significant correlations compared to the recent period. This could be due to the larger number of data points available for the longer period, which enhances the robustness of the findings. The early period from 1985 to 2011 shows the highest proportion of significant correlations, followed by the entire period, with the recent period showing a comparatively low share of significant values. This trend highlights the importance of data quantity and the potential impact of short-term fluctuations in more recent years.

Regarding the direction of the correlations, significant negative correlations dominate the entire period and the early period. In contrast, the recent period shows a more balanced ratio of positive and negative correlations. This shift may be explained by the plateauing of DDD-costs for many drugs since 2011, suggesting that other factors might become increasingly important in determining prescription trends.

In terms of the strength of the correlations, all three analyses display similar behavior, with a slightly higher number and ratio of strong correlations observed in the early period. The recent period, however, includes both positive and negative strong correlations, further supporting the hypothesis that the influence of DDD-costs was particularly high at the beginning of the recording period but remains significant in recent years.

## Limitations

The analysis in this study is based on data from the Arzneiverordnungsreport. As only outpatient prescriptions of the GKV system are included, no assessment can be made regarding prescriptions in hospitals or via private health insurance. As the data are based on developments in Germany, they are not directly transferable to other countries. No differentiation was made according to patient age or region of Germany.

Data collection for DDD-prescriptions and costs depended on the structure of the chapters under consideration in the Arzneiverordnungsreport. Changes in the structure of these sections over the years have led to biases in the data for clindamycin and phenoxymethylpenicillin in 2012 (Schwabe and Paffrath 2013) (see Figs. S5 and S7). Furthermore, the limited number of examined antibacterial drugs and the specific factors considered restrict the ability to generalize the findings. Other potential influencing factors were not included in the analysis, which may affect the study’s outcomes. As the study only considered DDD-costs as an influencing factor, it cannot make any statements about the influence of other factors on DDD-prescriptions.

The selection of the years analyzed also impacts the conclusions drawn. Different time periods may yield varying results, and the significance of the findings is influenced by the number of data points available. Since data were not available for all antibacterial drugs across all years, this might introduce uncertainties in comparisons. The relatively short period of 11 years for the 2012–2022 analysis further affects the significance of the correlations.

The study employed specific criteria for statistical methods, including the choice of statistical procedures and the thresholds for significant correlations at two-sided significance levels of 0.01 and 0.05. A strong correlation coefficient was determined as above ( ±) 0.8. Altering these parameters could lead to different conclusions.

## Conclusions and further perspectives

Previous examinations of our group have highlighted that the prescription of antibacterial drug use is influenced by certain political and economic events, with DDD-costs standing out as a major factor (Bindel and Seifert 2024). To substantiate this observation, further analyses were conducted to objectify the relationships of DDD-costs on DDD-prescriptions. The analysis spanned the entire period from 1985 to 2022, which was further divided into two sub-periods: 1985 to 2011 and 2012 to 2022.

Overall, DDD-costs exhibit a strong negative correlation with DDD-prescriptions across all time periods, evidenced by a high number and proportion of significant and strong total correlations. This persistent influence is particularly pronounced in the early period (1985–2011) and slightly diminishes in the recent period (2012–2022). This attenuation may due to the strong effects of the COVID pandemic and may indicate the rising importance of other factors influencing prescription trends, as most DDD-costs have plateaued since 2011 (see Fig. 2).

At the level of individual antibacterial drugs, a consistently strong influence of DDD-costs is observed for amoxicillin, amoxicillin clavulanic acid, and nitrofurantoin throughout the entire period (see Table 1). Notably, a larger proportion of the TOP5 drugs display this behavior, compared to the TOP6-15. Conversely, some drugs exhibit changing and unstable correlations between 2012–2022 versus the entire period and 1985–2011. In these cases, previous negative correlations between DDD-prescriptions and DDD-costs have shifted to positive correlations. When correlations are non-significant, their unreliability precludes meaningful assessment. However, significant changes in correlations for drugs like doxycycline, azithromycin, ciprofloxacin, and clarithromycin suggest evolving influencing factors for these specific drugs.

An association does not automatically imply causality (Mukaka 2012). With regard to the correlations of the TOP15 antibacterial drugs, however, this conclusion is plausible due to the predominantly strong correlation coefficients and high significance. In some cases, discrepancies arise due to distortions in prescription data, which might introduce uncertainties in comparisons. An example of this is the development of clindamycin and phenoxymethylpenicillin (see Fig. 1), which illustrates how important it is to have high-quality underlying data in order to recognize patterns and draw meaningful and comprehensive conclusions.

These findings have practical implications. It is concerning that costs may exert a greater influence on prescriptions than factors such as bacterial resistance development. Ideally, prescriptions should be based on drug efficacy rather than cost. However, this also presents an opportunity to influence prescriptions through cost regulation. Consideration could be given to whether price adjustments might effectively influence drug prescriptions and counteract adverse trends.

It becomes evident that a more concentrated effort should be made to implement systemic measures in addition to individual behavioral changes. Further investigations are necessary to confirm causality from the observed associations. With regard to other European countries, it is possible that DDD-costs could also have a strong influence on DDD-prescriptions, which is supported by the fact that the consumption of antibacterial substances in Germany and the EU-average have developed in the same line in recent years (ECDC 2024). For a definitive statement, it is necessary to analyze each country in detail. Extending the analysis to additional antibacterial drugs and further European countries could help validate and generalize these patterns. Moreover, future research should aim to examine the possible influencing factors that have gained importance in the development of DDD-prescriptions in recent years.

## Supplementary Information

Below is the link to the electronic supplementary material.

Supplementary file1 (DOCX 4496 KB)

## Data Availability

All source data for this study are available upon reasonable request from the authors.

## Abbreviations

AVR: Arzneiverordnungsreport (Drug Prescription Report regarding the GKV system of Germany)
AMNOG: Arzneimarktneuordnungsgesetz (Pharmaceutical Market Reorganization Act), 2011
DDD: Defined Daily Dose
GKV: Gesetzliche Krankenversicherung (statutory health insurance)
GMG: GKV-Modernisierungsgesetz (Statutory Health Insurance Modernization Act), 2004
RKI: Robert Koch Institute
SPSS: Statistical Product and Service Solutions (software for statistical analyses, IBM)
TOP15: Group of the 15 most prescribed antibacterial drugs (based on the year 2022)
TOP5: Group of the 5 most prescribed antibacterial drugs (based on the year 2022)
TOP6-15: Group of the sixth to 15th most prescribed antibacterial drugs (based on the year 2022)
