# Supplementary material for: DDD-costs have a strong influence on antibacterial drug prescription in Germany: a differentiated correlation analysis from 1985 to 2022
Source: Naunyn Schmiedebergs Arch Pharmacol. 2024 Jul 23;398(1):617–28. doi: 10.1007/s00210-024-03288-0 (PMC11787216; doi:10.1007/s00210-024-03288-0)
Supplement: Supplementary file 1 — Supplementary file1 (DOCX 4496 KB) [file 210_2024_3288_MOESM1_ESM.docx]

**DDD-costs have a strong influence on antibacterial drug prescription in Germany:**

**A differentiated correlation analysis from 1985 to 202**

**Lilly Josephine Bindel and Roland Seifert**

Supplemental Tables

***Table S1:*** *Correlation matrix, generated by SPSS, of the DDD-prescriptions and DDD-costs for amoxicillin from 1985-2022. Dark green colour and “**” indicates a significant correlation at the 0.01 level. Light green colour and “*” indicates a significant correlation at the 0.05 level. Orange colour indicates no significant correlation.*

|  | | 1 Amoxicillin  DDD-prescriptions | 1 Amoxicillin  DDD-costs |
| --- | --- | --- | --- |
| 1 Amoxicillin  DDD-prescriptions | Pearson Correlation | -- |  |
|  | N | 38 |  |
| 1 Amoxicillin  DDD-costs | Pearson Correlation | -0.941** | -- |
|  | Sig. (2-tailed) | < 0.001 |  |
|  | N | 38 | 38 |

***Table S2:*** *Correlation matrix, generated by SPSS, of the DDD-prescriptions and DDD-costs for amoxicillin from 1985-2011. Dark green colour and “**” indicates a significant correlation at the 0.01 level. Light green colour and “*” indicates a significant correlation at the 0.05 level. Orange colour indicates no significant correlation.*

|  | | 1 Amoxicillin DDD-prescriptions | 1 Amoxicillin DDD-costs |
| --- | --- | --- | --- |
| 1 Amoxicillin  DDD-prescriptions | Pearson Correlation | -- |  |
|  | N | 27 |  |
| 1 Amoxicillin  DDD-costs | Pearson Correlation | -0.956^**^ | -- |
|  | Sig. (2-tailed) | 0.000 |  |
|  | N | 27 | 27 |

***Table S3:*** *Correlation matrix, generated by SPSS, of the DDD-prescriptions and DDD-costs for amoxicillin from 1912-2022. Dark green colour and “**” indicates a significant correlation at the 0.01 level. Light green colour and “*” indicates a significant correlation at the 0.05 level. Orange colour indicates no significant correlation.*

|  | | 1 Amoxicillin DDD-prescriptions | 1 Amoxicillin DDD-costs |
| --- | --- | --- | --- |
| 1 Amoxicillin  DDD-prescriptions | Pearson Correlation | -- |  |
|  | N | 11 |  |
| 1 Amoxicillin  DDD-costs | Pearson Correlation | -0.915^**^ | -- |
|  | Sig. (2-tailed) | 0.000 |  |
|  | N | 11 | 11 |

***Table S4:*** *Correlation matrix, generated by SPSS, of the DDD-prescriptions and DDD-costs for cefuroxime axetil from 1990-2022. Dark green colour and “**” indicates a significant correlation at the 0.01 level. Light green colour and “*” indicates a significant correlation at the 0.05 level. Orange colour indicates no significant correlation.*

|  | | 2 Cefuroxime axetil DDD-prescriptions | 2 Cefuroxime axetil  DDD-costs |
| --- | --- | --- | --- |
| 2 Cefuroxime axetil  DDD-prescriptions | Pearson Correlation | -- |  |
|  | N | 33 |  |
| 2 Cefuroxime axetil  DDD-costs | Pearson Correlation | -0.900^**^ | -- |
|  | Sig. (2-tailed) | 0.000 |  |
|  | N | 33 | 33 |

***Table S5:*** *Correlation matrix, generated by SPSS, of the DDD-prescriptions and DDD-costs for cefuroxime axetil from 1990-2011. Dark green colour and “**” indicates a significant correlation at the 0.01 level. Light green colour and “*” indicates a significant correlation at the 0.05 level. Orange colour indicates no significant correlation.*

|  | | 2 Cefuroxime axetil DDD-prescriptions | 2 Cefuroxime axetil  DDD-costs |
| --- | --- | --- | --- |
| 2 Cefuroxime axetil  DDD-prescriptions | Pearson Correlation | -- |  |
|  | N | 22 |  |
| 2 Cefuroxime axetil  DDD-costs | Pearson Correlation | -0.911^**^ | -- |
|  | Sig. (2-tailed) | 0.000 |  |
|  | N | 22 | 22 |

***Table S6:*** *Correlation matrix, generated by SPSS, of the DDD-prescriptions and DDD-costs for cefuroxime axetil from 2012-2022. Dark green colour and “**” indicates a significant correlation at the 0.01 level. Light green colour and “*” indicates a significant correlation at the 0.05 level. Orange colour indicates no significant correlation.*

|  | | 2 Cefuroxime axetil DDD-prescriptions | 2 Cefuroxime axetil  DDD-costs |
| --- | --- | --- | --- |
| 2 Cefuroxime axetil  DDD-prescriptions | Pearson Correlation | -- |  |
|  | N | 11 |  |
| 2 Cefuroxime axetil  DDD-costs | Pearson Correlation | -0.272 | -- |
|  | Sig. (2-tailed) | 0.418 |  |
|  | N | 11 | 11 |

***Table S7:*** *Correlation matrix, generated by SPSS, of the DDD-prescriptions and DDD-costs for doxycycline from 1985-2022. Dark green colour and “**” indicates a significant correlation at the 0.01 level. Light green colour and “*” indicates a significant correlation at the 0.05 level. Orange colour indicates no significant correlation.*

|  | | 3 Doxycycline DDD-prescriptions | 3 Doxycycline DDD-costs |
| --- | --- | --- | --- |
| 3 Doxycycline  DDD-prescriptions | Pearson Correlation | -- |  |
|  | N | 38 |  |
| 3 Doxycycline  DDD-costs | Pearson Correlation | -0.287 | -- |
|  | Sig. (2-tailed) | 0.080 |  |
|  | N | 38 | 38 |

***Table S8:*** *Correlation matrix, generated by SPSS, of the DDD-prescriptions and DDD-costs for doxycycline from 1985-2011. Dark green colour and “**” indicates a significant correlation at the 0.01 level. Light green colour and “*” indicates a significant correlation at the 0.05 level. Orange colour indicates no significant correlation.*

|  | | 3 Doxycycline DDD-prescriptions | 3 Doxycycline DDD-costs |
| --- | --- | --- | --- |
| 3 Doxycycline  DDD-prescriptions | Pearson Correlation | -- |  |
|  | N | 27 |  |
| 3 Doxycycline  DDD-costs | Pearson Correlation | -0.377 | -- |
|  | Sig. (2-tailed) | 0.053 |  |
|  | N | 27 | 27 |

***Table S9:*** *Correlation matrix, generated by SPSS, of the DDD-prescriptions and DDD-costs for doxycycline from 2012-2022. Dark green colour and “**” indicates a significant correlation at the 0.01 level. Light green colour and “*” indicates a significant correlation at the 0.05 level. Orange colour indicates no significant correlation.*

|  | | 3 Doxycycline DDD-prescriptions | 3 Doxycycline DDD-costs |
| --- | --- | --- | --- |
| 3 Doxycycline  DDD-prescriptions | Pearson Correlation | -- |  |
|  | N | 11 |  |
| 3 Doxycycline  DDD-costs | Pearson Correlation | 0.722^*^ | -- |
|  | Sig. (2-tailed) | 0.012 |  |
|  | N | 11 | 11 |

***Table S10:*** *Correlation matrix, generated by SPSS, of the DDD-prescriptions and DDD-costs for amoxicillin clavulanic acid from 1986-2022. Dark green colour and “**” indicates a significant correlation at the 0.01 level. Light green colour and “*” indicates a significant correlation at the 0.05 level. Orange colour indicates no significant correlation.*

|  | | 4 Amoxicillin clavulanic acid DDD-prescriptions | 4 Amoxicillin clavulanic acid DDD-costs |
| --- | --- | --- | --- |
| 4 Amoxicillin clavulanic acid DDD-prescriptions | Pearson Correlation | -- |  |
|  | N | 37 |  |
| 4 Amoxicillin clavulanic acid DDD-costs | Pearson Correlation | -0.749** | -- |
|  | Sig. (2-tailed) | <.001 |  |
|  | N | 37 | 37 |

***Table S11:*** *Correlation matrix, generated by SPSS, of the DDD-prescriptions and DDD-costs for amoxicillin clavulanic acid from 1986-2011. Dark green colour and “**” indicates a significant correlation at the 0.01 level. Light green colour and “*” indicates a significant correlation at the 0.05 level. Orange colour indicates no significant correlation.*

|  | | 4 Amoxicillin clavulanic acid DDD-prescriptions | 4 Amoxicillin clavulanic acid DDD-costs |
| --- | --- | --- | --- |
| 4 Amoxicillin clavulanic acid DDD-prescriptions | Pearson Correlation | -- |  |
|  | N | 26 |  |
| 4 Amoxicillin clavulanic acid DDD-costs | Pearson Correlation | -0.878^**^ | -- |
|  | Sig. (2-tailed) | 0.000 |  |
|  | N | 26 | 26 |

***Table S12:*** *Correlation matrix, generated by SPSS, of the DDD-prescriptions and DDD-costs for amoxicillin clavulanic acid from 2012-2022. Dark green colour and “**” indicates a significant correlation at the 0.01 level. Light green colour and “*” indicates a significant correlation at the 0.05 level. Orange colour indicates no significant correlation.*

|  | | 4 Amoxicillin clavulanic acid DDD-prescriptions | 4 Amoxicillin clavulanic acid DDD-costs |
| --- | --- | --- | --- |
| 4 Amoxicillin clavulanic acid DDD-prescriptions | Pearson Correlation | -- |  |
|  | N | 11 |  |
| 4 Amoxicillin clavulanic acid DDD-costs | Pearson Correlation | -0.912^**^ | -- |
|  | Sig. (2-tailed) | 0.000 |  |
|  | N | 11 | 11 |

***Table S13:*** *Correlation matrix, generated by SPSS, of the DDD-prescriptions and DDD-costs for clindamycin from 1986-2022. Dark green colour and “**” indicates a significant correlation at the 0.01 level. Light green colour and “*” indicates a significant correlation at the 0.05 level. Orange colour indicates no significant correlation.*

|  | | 5 Clindamycin DDD-prescriptions | 5 Clindamycin DDD-costs |
| --- | --- | --- | --- |
| 5 Clindamycin  DDD-prescriptions | Pearson Correlation | -- |  |
|  | N | 37 |  |
| 5 Clindamycin  DDD-costs | Pearson Correlation | -0.800** | -- |
|  | Sig. (2-tailed) | <.001 |  |
|  | N | 37 | 37 |

***Table S14:*** *Correlation matrix, generated by SPSS, of the DDD-prescriptions and DDD-costs for clindamycin from 1986-2011. Dark green colour and “**” indicates a significant correlation at the 0.01 level. Light green colour and “*” indicates a significant correlation at the 0.05 level. Orange colour indicates no significant correlation.*

|  | | 5 Clindamycin DDD-prescriptions | 5 Clindamycin DDD-costs |
| --- | --- | --- | --- |
| 5 Clindamycin  DDD-prescriptions | Pearson Correlation | -- |  |
|  | N | 26 |  |
| 5 Clindamycin  DDD-costs | Pearson Correlation | -0.897^**^ | -- |
|  | Sig. (2-tailed) | 0.000 |  |
|  | N | 26 | 26 |

***Table S15:*** *Correlation matrix, generated by SPSS, of the DDD-prescriptions and DDD-costs for clindamycin from 2012-2022. Dark green colour and “**” indicates a significant correlation at the 0.01 level. Light green colour and “*” indicates a significant correlation at the 0.05 level. Orange colour indicates no significant correlation.*

|  | | 5 Clindamycin DDD-prescriptions | 5 Clindamycin DDD-costs |
| --- | --- | --- | --- |
| 5 Clindamycin  DDD-prescriptions | Pearson Correlation | -- |  |
|  | N | 11 |  |
| 5 Clindamycin  DDD-costs | Pearson Correlation | 0.448 | -- |
|  | Sig. (2-tailed) | 0.167 |  |
|  | N | 11 | 11 |

***Table S16:*** *Correlation matrix, generated by SPSS, of the DDD-prescriptions and DDD-costs for azithromycin from 1994-2022. Dark green colour and “**” indicates a significant correlation at the 0.01 level. Light green colour and “*” indicates a significant correlation at the 0.05 level. Orange colour indicates no significant correlation.*

|  | | 6 Azithromycin DDD-prescriptions | 6 Azithromycin DDD-costs |
| --- | --- | --- | --- |
| 6 Azithromycin DDD-prescriptions | Pearson Correlation | -- |  |
|  | N | 29 |  |
| 6 Azithromycin DDD-costs | Pearson Correlation | -0.719^**^ | -- |
|  | Sig. (2-tailed) | 0.000 |  |
|  | N | 29 | 29 |

***Table S17:*** *Correlation matrix, generated by SPSS, of the DDD-prescriptions and DDD-costs for azithromycin from 1994-2011. Dark green colour and “**” indicates a significant correlation at the 0.01 level. Light green colour and “*” indicates a significant correlation at the 0.05 level. Orange colour indicates no significant correlation.*

|  | | 6 Azithromycin DDD-prescriptions | 6 Azithromycin DDD-costs |
| --- | --- | --- | --- |
| 6 Azithromycin  DDD-prescriptions | Pearson Correlation | -- |  |
|  | N | 18 |  |
| 6 Azithromycin  DDD-costs | Pearson Correlation | -0.590^**^ | -- |
|  | Sig. (2-tailed) | 0.010 |  |
|  | N | 18 | 18 |

***Table S18:*** *Correlation matrix, generated by SPSS, of the DDD-prescriptions and DDD-costs for azithromycin from 2012-2022. Dark green colour and “**” indicates a significant correlation at the 0.01 level. Light green colour and “*” indicates a significant correlation at the 0.05 level. Orange colour indicates no significant correlation.*

|  | | 6 Azithromycin DDD-prescriptions | 6 Azithromycin DDD-costs |
| --- | --- | --- | --- |
| 6 Azithromycin  DDD-prescriptions | Pearson Correlation | -- |  |
|  | N | 11 |  |
| 6 Azithromycin  DDD-costs | Pearson Correlation | 0.747^**^ | -- |
|  | Sig. (2-tailed) | 0.008 |  |
|  | N | 11 | 11 |

***Table S19:*** *Correlation matrix, generated by SPSS, of the DDD-prescriptions and DDD-costs for phenoxymethylpenicillin from 1985-2022. Dark green colour and “**” indicates a significant correlation at the 0.01 level. Light green colour and “*” indicates a significant correlation at the 0.05 level. Orange colour indicates no significant correlation.*

|  | | 7 Phenoxymethylpenicillin DDD-prescriptions | 7 Phenoxymethylpenicillin DDD-costs |
| --- | --- | --- | --- |
| 7 Phenoxymethylpenicillin DDD-prescriptions | Pearson Correlation | -- |  |
|  | N | 38 |  |
| 7 Phenoxymethylpenicillin DDD-costs | Pearson Correlation | -0.543^**^ | -- |
|  | Sig. (2-tailed) | 0.000 |  |
|  | N | 38 | 38 |

***Table S20:*** *Correlation matrix, generated by SPSS, of the DDD-prescriptions and DDD-costs for phenoxymethylpenicillin from 1985-2011. Dark green colour and “**” indicates a significant correlation at the 0.01 level. Light green colour and “*” indicates a significant correlation at the 0.05 level. Orange colour indicates no significant correlation.*

|  | | 7 Phenoxymethylpenicillin DDD-prescriptions | 7 Phenoxymethylpenicillin DDD-costs |
| --- | --- | --- | --- |
| 7 Phenoxymethylpenicillin DDD-prescription | Pearson Correlation | -- |  |
|  | N | 27 |  |
| 7 Phenoxymethylpenicillin DDD-costs | Pearson Correlation | -0.634^**^ | -- |
|  | Sig. (2-tailed) | 0.000 |  |
|  | N | 27 | 27 |

***Table S21:*** *Correlation matrix, generated by SPSS, of the DDD-prescriptions and DDD-costs for phenoxymethylpenicillin from 2012-2022. Dark green colour and “**” indicates a significant correlation at the 0.01 level. Light green colour and “*” indicates a significant correlation at the 0.05 level. Orange colour indicates no significant correlation.*

|  | | 7 Phenoxymethylpenicillin DDD-prescriptions | 7 Phenoxymethylpenicillin DDD-costs |
| --- | --- | --- | --- |
| 7 Phenoxymethylpenicillin DDD-prescriptions | Pearson Correlation | -- |  |
|  | N | 11 |  |
| 7 Phenoxymethylpenicillin DDD-costs | Pearson Correlation | 0.029 | -- |
|  | Sig. (2-tailed) | 0.932 |  |
|  | N | 11 | 11 |

***Table S22:*** *Correlation matrix, generated by SPSS, of the DDD-prescriptions and DDD-costs for sulfamethoxazole-trimethoprim from 1985-2022. Dark green colour and “**” indicates a significant correlation at the 0.01 level. Light green colour and “*” indicates a significant correlation at the 0.05 level. Orange colour indicates no significant correlation.*

|  | | 8 Sulfamethoxazole-Trimethoprim DDD-prescriptions | 8 Sulfamethoxazole-Trimethoprim DDD-costs |
| --- | --- | --- | --- |
| 8 Sulfamethoxazole-Trimethoprim  DDD-prescriptions | Pearson Correlation | -- |  |
|  | N | 38 |  |
| 8 Sulfamethoxazole-Trimethoprim  DDD-costs | Pearson Correlation | -0.758^**^ | -- |
|  | Sig. (2-tailed) | 0.000 |  |
|  | N | 38 | 38 |

***Table S23:*** *Correlation matrix, generated by SPSS, of the DDD-prescriptions and DDD-costs for sulfamethoxazole-trimethoprim from 1985-2011. Dark green colour and “**” indicates a significant correlation at the 0.01 level. Light green colour and “*” indicates a significant correlation at the 0.05 level. Orange colour indicates no significant correlation.*

|  | | 8 Sulfamethoxazole-Trimethoprim DDD-prescriptions | 8 Sulfamethoxazole-Trimethoprim DDD-costs |
| --- | --- | --- | --- |
| 8 Sulfamethoxazole-Trimethoprim  DDD-prescriptions | Pearson Correlation | -- |  |
|  | N | 27 |  |
| 8 Sulfamethoxazole-Trimethoprim DDD-costs | Pearson Correlation | -0.658^**^ | -- |
|  | Sig. (2-tailed) | 0.000 |  |
|  | N | 27 | 27 |

***Table S24:*** *Correlation matrix, generated by SPSS, of the DDD-prescriptions and DDD-costs for sulfamethoxazole-trimethoprim from 2012-2022. Dark green colour and “**” indicates a significant correlation at the 0.01 level. Light green colour and “*” indicates a significant correlation at the 0.05 level. Orange colour indicates no significant correlation.*

|  | | 8 Sulfamethoxazole-Trimethoprim DDD-prescriptions | 8 Sulfamethoxazole-Trimethoprim DDD-costs |
| --- | --- | --- | --- |
| 8 Sulfamethoxazole-Trimethoprim  DDD-prescriptions | Pearson Correlation | -- |  |
|  | N | 11 |  |
| 8 Sulfamethoxazole-Trimethoprim  DDD-costs | Pearson Correlation | 0.193 | -- |
|  | Sig. (2-tailed) | 0.570 |  |
|  | N | 11 | 11 |

***Table S25:*** *Correlation matrix, generated by SPSS, of the DDD-prescriptions and DDD-costs for nitrofurantoin from 2007-2022. Dark green colour and “**” indicates a significant correlation at the 0.01 level. Light green colour and “*” indicates a significant correlation at the 0.05 level. Orange colour indicates no significant correlation.*

|  | | 9 Nitrofurantoin DDD-prescriptions | 9 Nitrofurantoin DDD-costs |
| --- | --- | --- | --- |
| 9 Nitrofurantoin  DDD-prescriptions | Pearson Correlation | -- |  |
|  | N | 16 |  |
| 9 Nitrofurantoin  DDD-costs | Pearson Correlation | -0.895^**^ | -- |
|  | Sig. (2-tailed) | 0.000 |  |
|  | N | 16 | 16 |

***Table S26:*** *Correlation matrix, generated by SPSS, of the DDD-prescriptions and DDD-costs for nitrofurantoin from 2007-2011. Dark green colour and “**” indicates a significant correlation at the 0.01 level. Light green colour and “*” indicates a significant correlation at the 0.05 level. Orange colour indicates no significant correlation.*

|  | | 9 Nitrofurantoin DDD-prescriptions | 9 Nitrofurantoin DDD-costs |
| --- | --- | --- | --- |
| 9 Nitrofurantoin  DDD-prescriptions | Pearson Correlation | -- |  |
|  | N | 5 |  |
| 9 Nitrofurantoin  DDD-costs | Pearson Correlation | -0.920^*^ | -- |
|  | Sig. (2-tailed) | 0.027 |  |
|  | N | 5 | 5 |

***Table S27:*** *Correlation matrix, generated by SPSS, of the DDD-prescriptions and DDD-costs for nitrofurantoin from 2011-2022. Dark green colour and “**” indicates a significant correlation at the 0.01 level. Light green colour and “*” indicates a significant correlation at the 0.05 level. Orange colour indicates no significant correlation.*

|  | | 9 Nitrofurantoin DDD-prescriptions | 9 Nitrofurantoin DDD-costs |
| --- | --- | --- | --- |
| 9 Nitrofurantoin  DDD-prescriptions | Pearson Correlation | -- |  |
|  | N | 11 |  |
| 9 Nitrofurantoin  DDD-costs | Pearson Correlation | -0.879^**^ | -- |
|  | Sig. (2-tailed) | 0.000 |  |
|  | N | 11 | 11 |

***Table S28:*** *Correlation matrix, generated by SPSS, of the DDD-prescriptions and DDD-costs for ciprofloxacin from 1987-2022. Dark green colour and “**” indicates a significant correlation at the 0.01 level. Light green colour and “*” indicates a significant correlation at the 0.05 level. Orange colour indicates no significant correlation.*

|  | | 10 Ciprofloxacin DDD-prescriptions | 10 Ciprofloxacin DDD-costs |
| --- | --- | --- | --- |
| 10 Ciprofloxacin  DDD-prescriptions | Pearson Correlation | -- |  |
|  | N | 36 |  |
| 10 Ciprofloxacin  DDD-costs | Pearson Correlation | -0.533^**^ | -- |
|  | Sig. (2-tailed) | 0.001 |  |
|  | N | 36 | 36 |

***Table S29:*** *Correlation matrix, generated by SPSS, of the DDD-prescriptions and DDD-costs for ciprofloxacin from 1987-2011. Dark green colour and “**” indicates a significant correlation at the 0.01 level. Light green colour and “*” indicates a significant correlation at the 0.05 level. Orange colour indicates no significant correlation.*

|  | | 10 Ciprofloxacin DDD-prescriptions | 10 Ciprofloxacin DDD-costs |
| --- | --- | --- | --- |
| 10 Ciprofloxacin  DDD-prescriptions | Pearson Correlation | -- |  |
|  | N | 25 |  |
| 10 Ciprofloxacin  DDD-costs | Pearson Correlation | -0.476^*^ | -- |
|  | Sig. (2-tailed) | 0.016 |  |
|  | N | 25 | 25 |

***Table S30:*** *Correlation matrix, generated by SPSS, of the DDD-prescriptions and DDD-costs for ciprofloxacin from 2012-2022. Dark green colour and “**” indicates a significant correlation at the 0.01 level. Light green colour and “*” indicates a significant correlation at the 0.05 level. Orange colour indicates no significant correlation.*

|  | | 10 Ciprofloxacin DDD-prescriptions | 10 Ciprofloxacin DDD-costs |
| --- | --- | --- | --- |
| 10 Ciprofloxacin  DDD-prescriptions | Pearson Correlation | -- |  |
|  | N | 11 |  |
| 10 Ciprofloxacin  DDD-costs | Pearson Correlation | 0.950^**^ | -- |
|  | Sig. (2-tailed) | 0.000 |  |
|  | N | 11 | 11 |

***Table S31:*** *Correlation matrix, generated by SPSS, of the DDD-prescriptions and DDD-costs for clarithromycin from 1991-2022. Dark green colour and “**” indicates a significant correlation at the 0.01 level. Light green colour and “*” indicates a significant correlation at the 0.05 level. Orange colour indicates no significant correlation.*

|  | | 11 Clarithromycin DDD-prescriptions | 11 Clarithromycin DDD-costs |
| --- | --- | --- | --- |
| 11 Clarithromycin  DDD-prescriptions | Pearson Correlation | -- |  |
|  | N | 32 |  |
| 11 Clarithromycin  DDD-costs | Pearson Correlation | -0.396^*^ | -- |
|  | Sig. (2-tailed) | 0.025 |  |
|  | N | 32 | 32 |

***Table S32:*** *Correlation matrix, generated by SPSS, of the DDD-prescriptions and DDD-costs for clarithromycin from 1991-2011. Dark green colour and “**” indicates a significant correlation at the 0.01 level. Light green colour and “*” indicates a significant correlation at the 0.05 level. Orange colour indicates no significant correlation.*

|  | | 11 Clarithromycin DDD-prescriptions | 11 Clarithromycin DDD-costs |
| --- | --- | --- | --- |
| 11 Clarithromycin  DDD-prescriptions | Pearson Correlation | -- |  |
|  | N | 21 |  |
| 11 Clarithromycin  DDD-costs | Pearson Correlation | -0.809^**^ | -- |
|  | Sig. (2-tailed) | 0.000 |  |
|  | N | 21 | 21 |

***Table S33:*** *Correlation matrix, generated by SPSS, of the DDD-prescriptions and DDD-costs for clarithromycin from 2012-2022. Dark green colour and “**” indicates a significant correlation at the 0.01 level. Light green colour and “*” indicates a significant correlation at the 0.05 level. Orange colour indicates no significant correlation.*

|  | | 11 Clarithromycin DDD-prescriptions | 11 Clarithromycin DDD-costs |
| --- | --- | --- | --- |
| 11 Clarithromycin  DDD-prescriptions | Pearson Correlation | -- |  |
|  | N | 11 |  |
| 11 Clarithromycin  DDD-costs | Pearson Correlation | 0.962^**^ | -- |
|  | Sig. (2-tailed) | 0.000 |  |
|  | N | 11 | 11 |

***Table S34:*** *Correlation matrix, generated by SPSS, of the DDD-prescriptions and DDD-costs for cefaclor from 1985-2022. Dark green colour and “**” indicates a significant correlation at the 0.01 level. Light green colour and “*” indicates a significant correlation at the 0.05 level. Orange colour indicates no significant correlation.*

|  | | 12 Cefaclor  DDD-prescriptions | 12 Cefaclor  DDD-costs |
| --- | --- | --- | --- |
| 12 Cefaclor  DDD-prescriptions | Pearson Correlation | -- |  |
|  | N | 38 |  |
| 12 Cefaclor  DDD-costs | Pearson Correlation | -0.819^**^ | -- |
|  | Sig. (2-tailed) | 0.000 |  |
|  | N | 38 | 38 |

***Table S35:*** *Correlation matrix, generated by SPSS, of the DDD-prescriptions and DDD-costs for cefaclor from 1985-2011. Dark green colour and “**” indicates a significant correlation at the 0.01 level. Light green colour and “*” indicates a significant correlation at the 0.05 level. Orange colour indicates no significant correlation.*

|  | | 12 Cefaclor  DDD-prescriptions | 12 Cefaclor  DDD-costs |
| --- | --- | --- | --- |
| 12 Cefaclor  DDD-prescriptions | Pearson Correlation | -- |  |
|  | N | 27 |  |
| 12 Cefaclor  DDD-costs | Pearson Correlation | -0.874^**^ | -- |
|  | Sig. (2-tailed) | 0.000 |  |
|  | N | 27 | 27 |

***Table S36:*** *Correlation matrix, generated by SPSS, of the DDD-prescriptions and DDD-costs for cefaclor from 2012-2022. Dark green colour and “**” indicates a significant correlation at the 0.01 level. Light green colour and “*” indicates a significant correlation at the 0.05 level. Orange colour indicates no significant correlation.*

|  | | 12 Cefaclor  DDD-prescriptions | 12 Cefaclor  DDD-costs |
| --- | --- | --- | --- |
| 12 Cefaclor  DDD-prescriptions | Pearson Correlation | -- |  |
|  | N | 11 |  |
| 12 Cefaclor  DDD-costs | Pearson Correlation | 0.415 | -- |
|  | Sig. (2-tailed) | 0.204 |  |
|  | N | 11 | 11 |

***Table S37:*** *Correlation matrix, generated by SPSS, of the DDD-prescriptions and DDD-costs for cefpodoxime from 1992-2022. Dark green colour and “**” indicates a significant correlation at the 0.01 level. Light green colour and “*” indicates a significant correlation at the 0.05 level. Orange colour indicates no significant correlation.*

|  | | 13 Cefpodoxime DDD-prescriptions | 13 Cefpodoxime DDD-costs |
| --- | --- | --- | --- |
| 13 Cefpodoxime  DDD-prescriptions | Pearson Correlation | -- |  |
|  | N | 31 |  |
| 13 Cefpodoxime  DDD-costs | Pearson Correlation | -0.787^**^ | -- |
|  | Sig. (2-tailed) | 0.000 |  |
|  | N | 31 | 31 |

***Table S38:*** *Correlation matrix, generated by SPSS, of the DDD-prescriptions and DDD-costs for cefpodoxime from 1992-2011. Dark green colour and “**” indicates a significant correlation at the 0.01 level. Light green colour and “*” indicates a significant correlation at the 0.05 level. Orange colour indicates no significant correlation.*

|  | | 13 Cefpodoxime DDD-prescriptions | 13 Cefpodoxime DDD-costs |
| --- | --- | --- | --- |
| 13 Cefpodoxime  DDD-prescriptions | Pearson Correlation | -- |  |
|  | N | 20 |  |
| 13 Cefpodoxime  DDD-costs | Pearson Correlation | -0.617^**^ | -- |
|  | Sig. (2-tailed) | 0.004 |  |
|  | N | 20 | 20 |

***Table S39:*** *Correlation matrix, generated by SPSS, of the DDD-prescriptions and DDD-costs for cefpodoxime from 2012-2022. Dark green colour and “**” indicates a significant correlation at the 0.01 level. Light green colour and “*” indicates a significant correlation at the 0.05 level. Orange colour indicates no significant correlation.*

|  | | 13 Cefpodoxime DDD-prescriptions | 13 Cefpodoxime DDD-costs |
| --- | --- | --- | --- |
| 13 Cefpodoxime  DDD-prescriptions | Pearson Correlation | -- |  |
|  | N | 11 |  |
| 13 Cefpodoxime  DDD-costs | Pearson Correlation | 0.537 | -- |
|  | Sig. (2-tailed) | 0.088 |  |
|  | N | 11 | 11 |

***Table S40:*** *Correlation matrix, generated by SPSS, of the DDD-prescriptions and DDD-costs for pivmecillinam from 2017-2022. Dark green colour and “**” indicates a significant correlation at the 0.01 level. Light green colour and “*” indicates a significant correlation at the 0.05 level. Orange colour indicates no significant correlation.*

|  | | 14 Pivmecillinam DDD-prescriptions | 14 Pivmecillinam DDD-costs |
| --- | --- | --- | --- |
| 14 Pivmecillinam  DDD-prescriptions | Pearson Correlation | -- |  |
|  | N | 6 |  |
| 14 Pivmecillinam  DDD-costs | Pearson Correlation | 0.761 | -- |
|  | Sig. (2-tailed) | 0.079 |  |
|  | N | 6 | 6 |

***Table S41:*** *Correlation matrix, generated by SPSS, of the DDD-prescriptions and DDD-costs for roxithromycin from 1991-2022. Dark green colour and “**” indicates a significant correlation at the 0.01 level. Light green colour and “*” indicates a significant correlation at the 0.05 level. Orange colour indicates no significant correlation.*

|  | | 15 Roxithromycin DDD-prescriptions | 15 Roxithromycin DDD-costs |
| --- | --- | --- | --- |
| 15 Roxithromycin  DDD-prescriptions | Pearson Correlation | -- |  |
|  | N | 32 |  |
| 15 Roxithromycin  DDD-costs | Pearson Correlation | 0.496^**^ | -- |
|  | Sig. (2-tailed) | 0.004 |  |
|  | N | 32 | 32 |

***Table S42:*** *Correlation matrix, generated by SPSS, of the DDD-prescriptions and DDD-costs for roxithromycin from 1991-2011. Dark green colour and “**” indicates a significant correlation at the 0.01 level. Light green colour and “*” indicates a significant correlation at the 0.05 level. Orange colour indicates no significant correlation.*

|  | | 15 Roxithromycin DDD-prescriptions | 15 Roxithromycin DDD-costs |
| --- | --- | --- | --- |
| 15 Roxithromycin  DDD-prescriptions | Pearson Correlation | -- |  |
|  | N | 21 |  |
| 15 Roxithromycin  DDD-costs | Pearson Correlation | -0.069 | -- |
|  | Sig. (2-tailed) | 0.765 |  |
|  | N | 21 | 21 |

***Table S43:*** *Correlation matrix, generated by SPSS, of the DDD-prescriptions and DDD-costs for roxithromycin from 2012-2022. Dark green colour and “**” indicates a significant correlation at the 0.01 level. Light green colour and “*” indicates a significant correlation at the 0.05 level. Orange colour indicates no significant correlation.*

|  | | 15 Roxithromycin DDD-prescriptions | 15 Roxithromycin DDD-costs |
| --- | --- | --- | --- |
| 15 Roxithromycin  DDD-prescriptions | Pearson Correlation | -- |  |
|  | N | 11 |  |
| 15 Roxithromycin  DDD-costs | Pearson Correlation | 0.009 | -- |
|  | Sig. (2-tailed) | 0.979 |  |
|  | N | 11 | 11 |

Supplemental Figures

***Fig. S1:*** *Development of the DDD-prescriptions and DDD-costs for amoxicillin.*


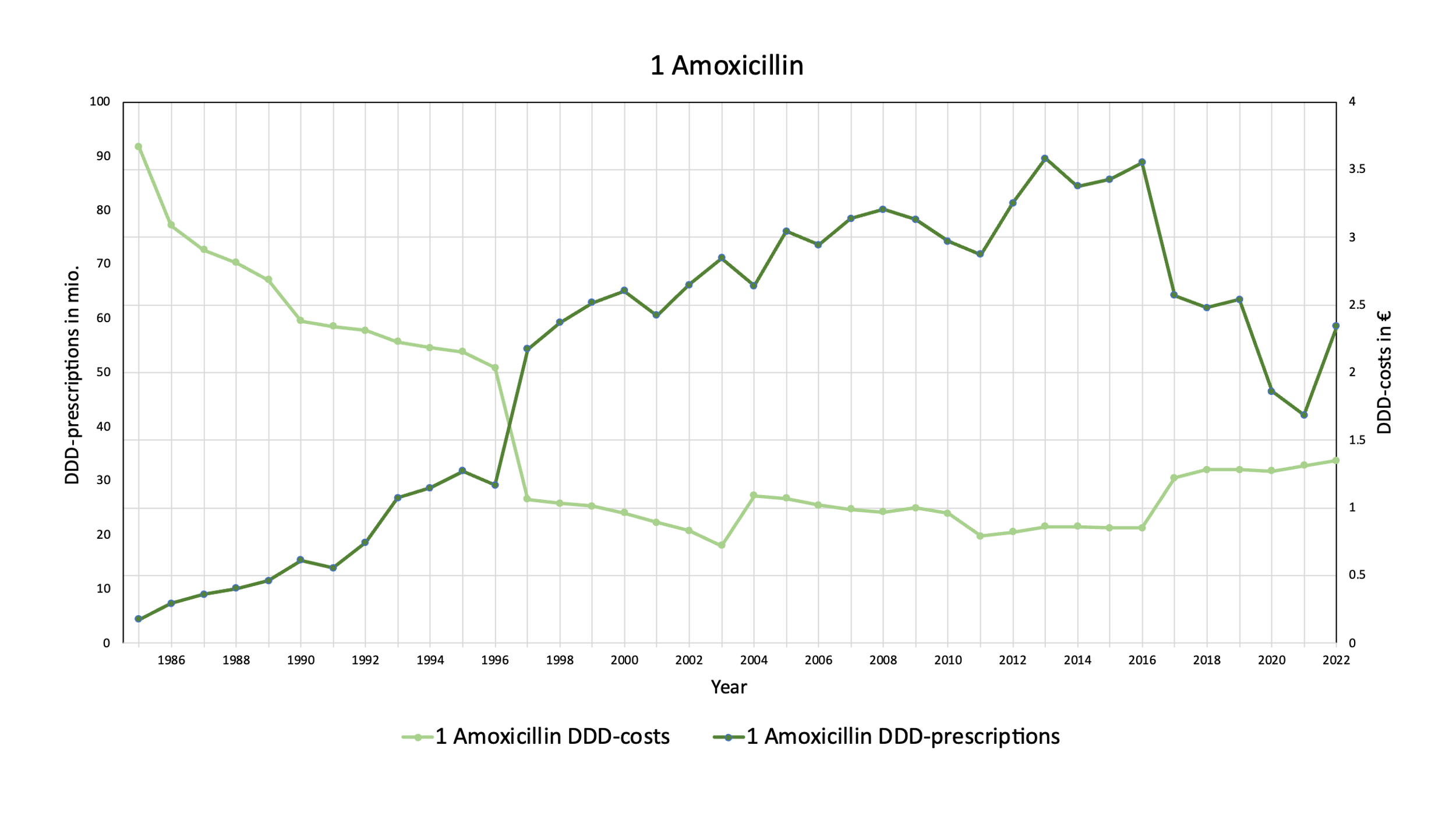


***Fig. S2:*** *Development of the DDD-prescriptions and DDD-costs for cefuroxime axetil.*


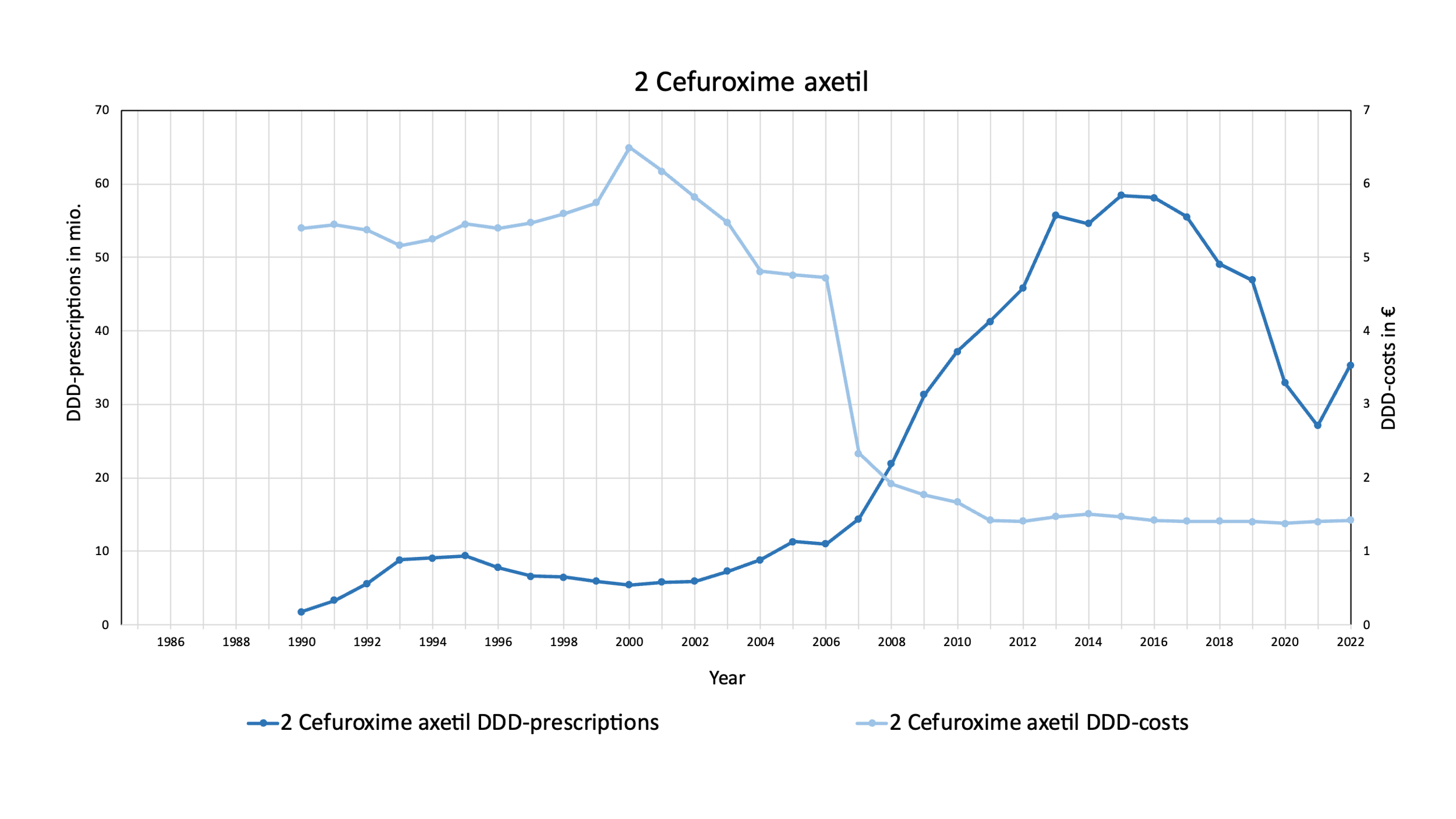


***Fig. S3:*** *Development of the DDD-prescriptions and DDD-costs for doxycycline.*


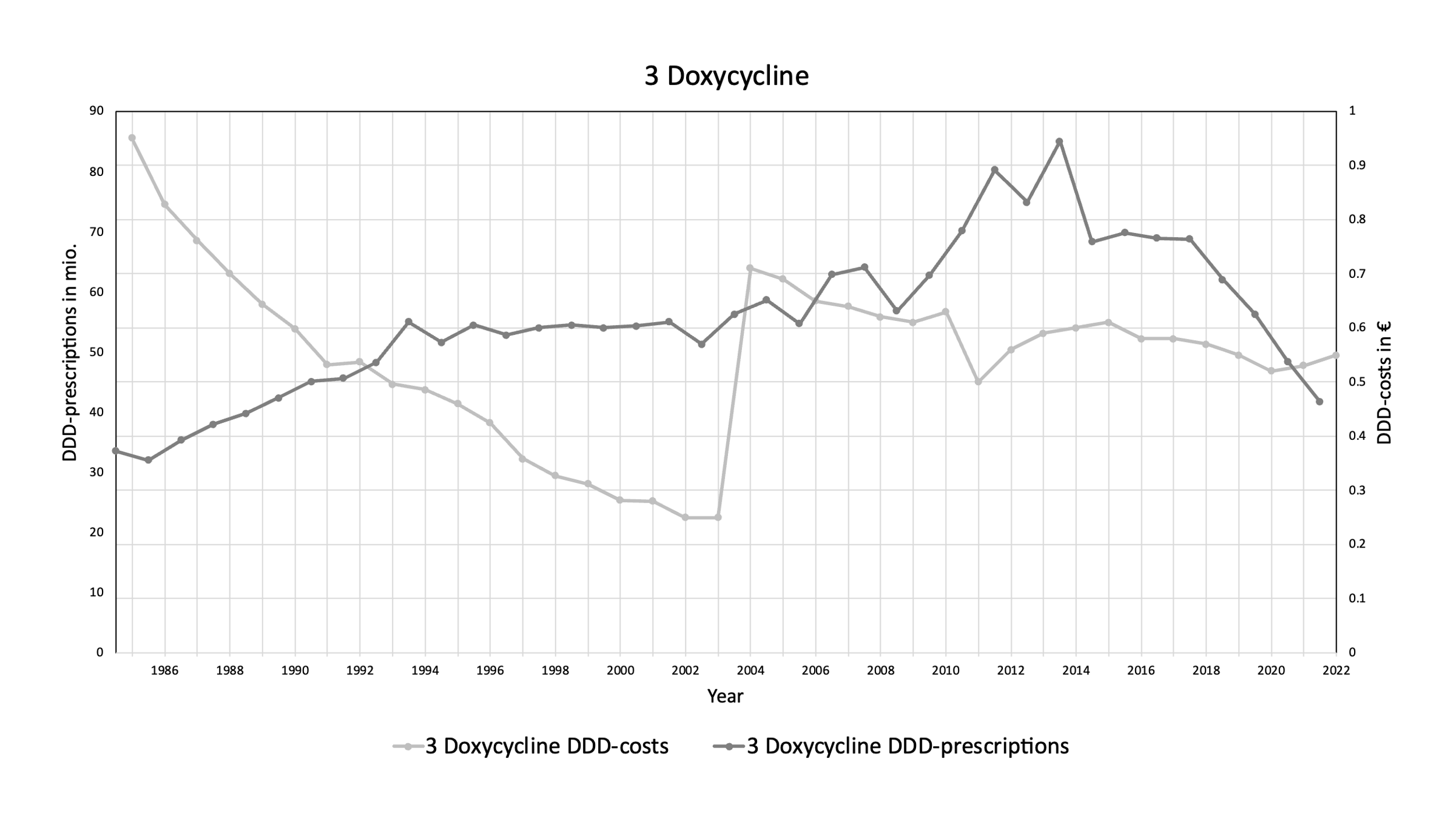


***Fig. S4:*** *Development of the DDD-prescriptions and DDD-costs for amoxicillin clavulanic acid.*


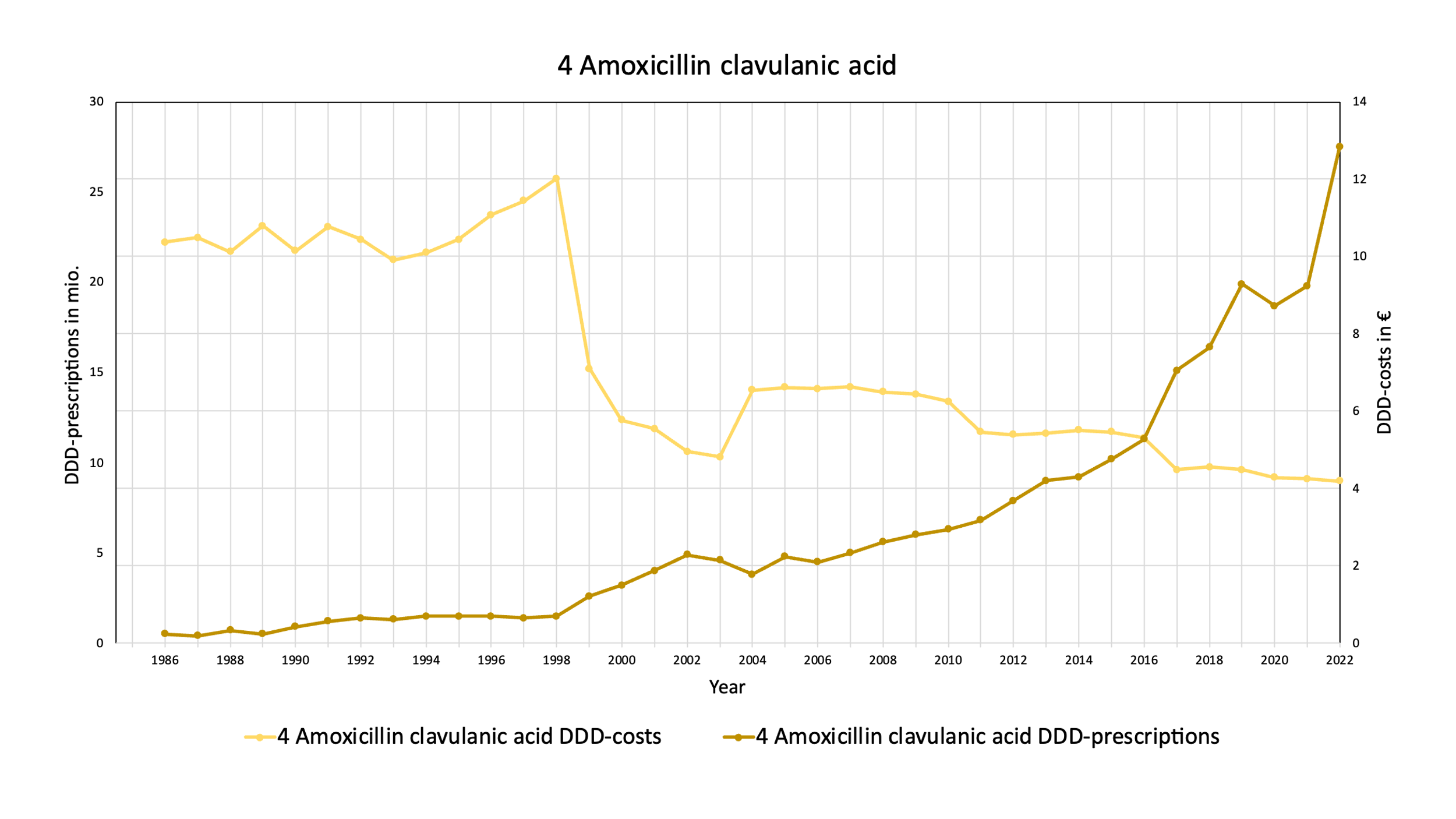


***Fig. S5:*** *Development of the DDD-prescriptions and DDD-costs for clindamycin.*


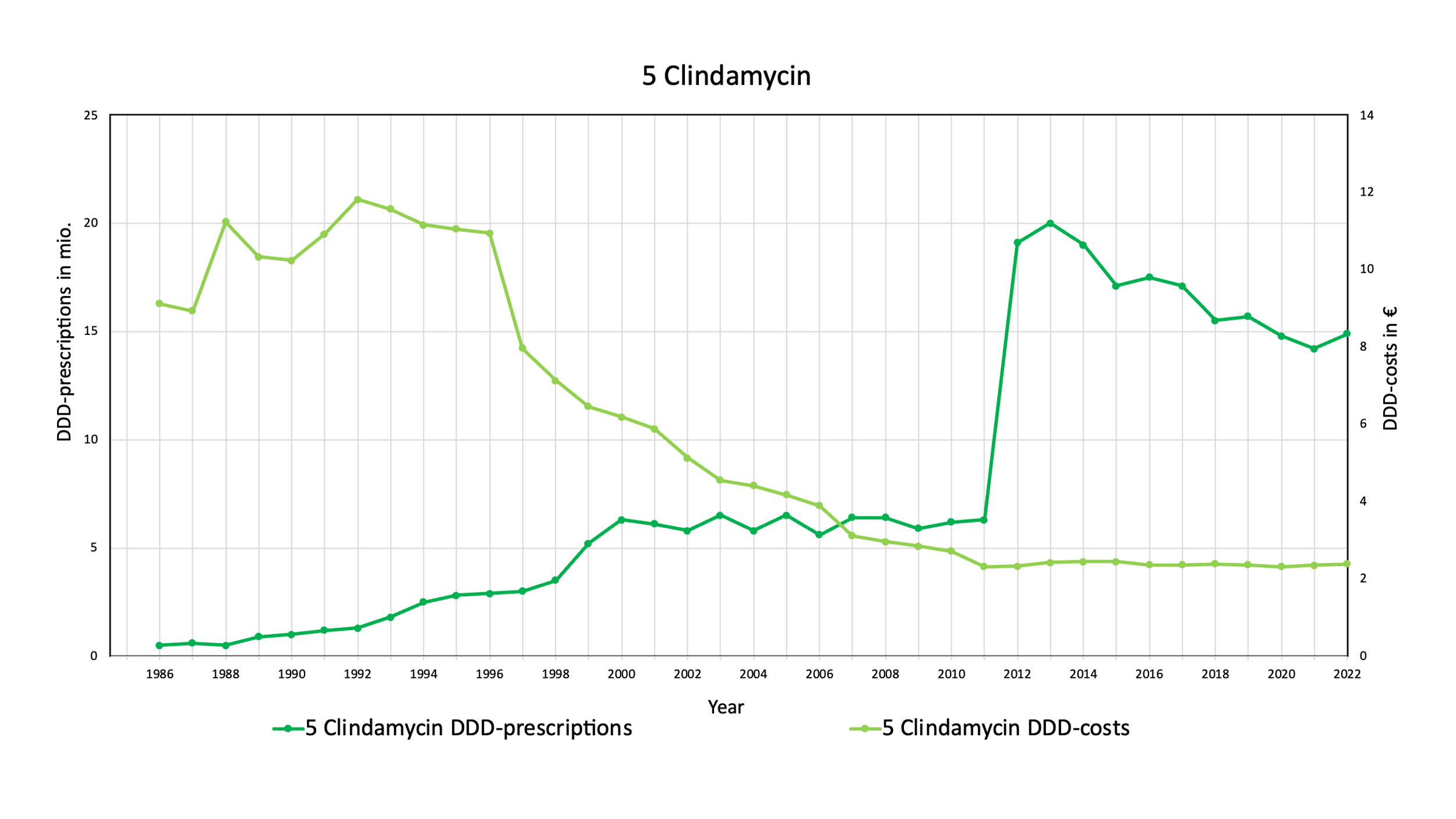


***Fig. S6:*** *Development of the DDD-prescriptions and DDD-costs for azithromycin.*


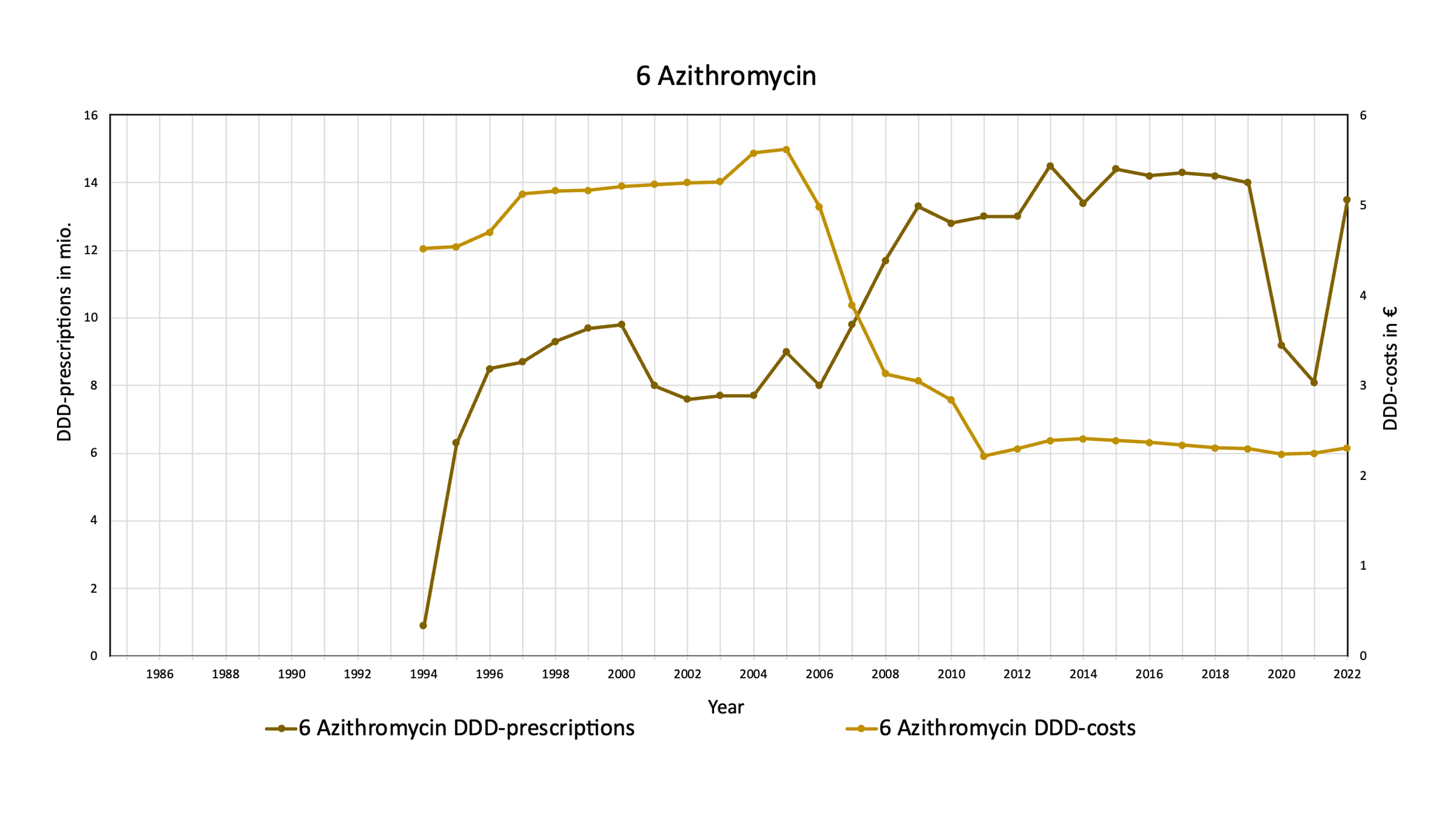


***Fig. S7:*** *Development of the DDD-prescriptions and DDD-costs for phenoxymethylpenicillin.*


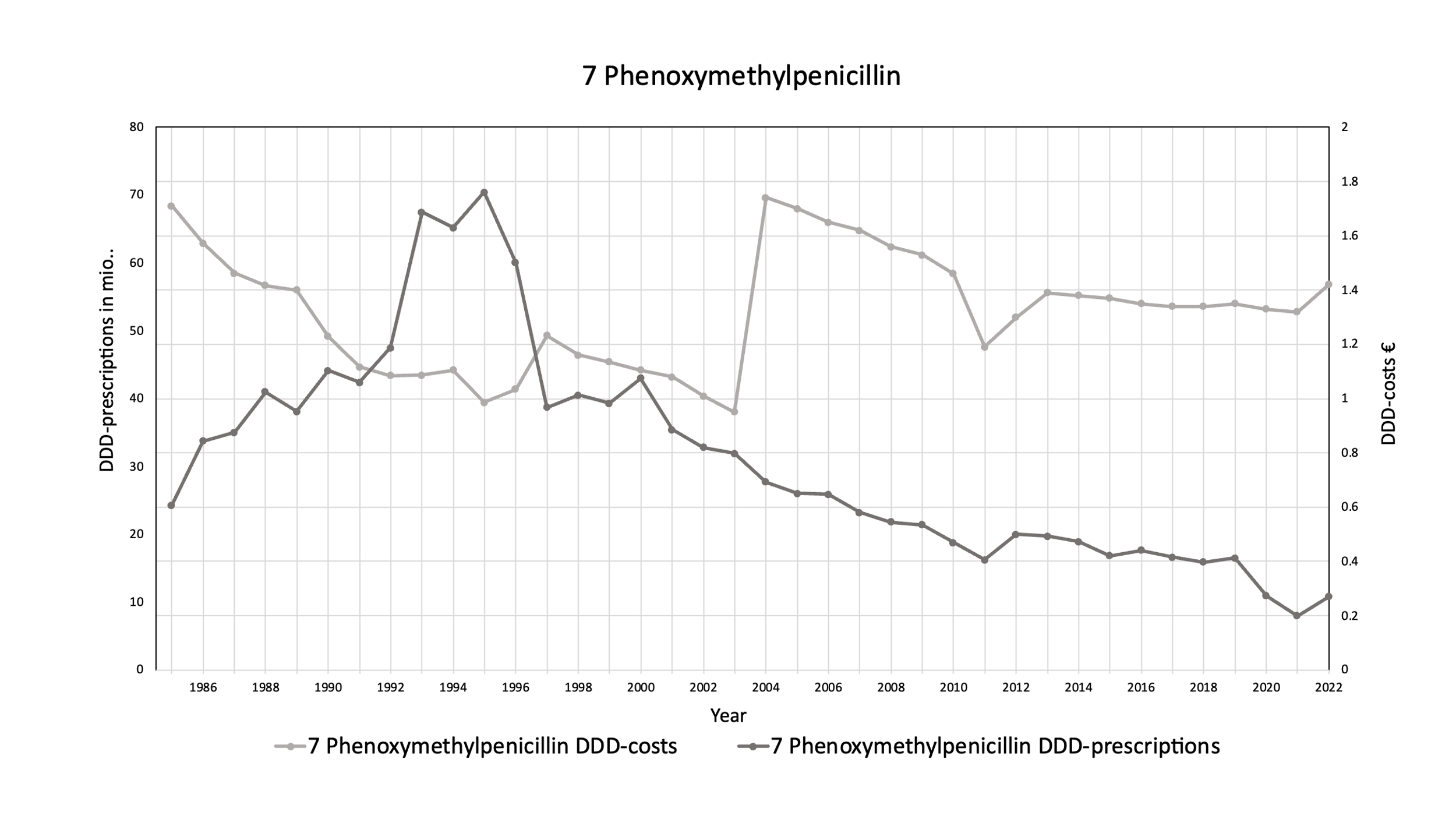


***Fig. S8:*** *Development of the DDD-prescriptions and DDD-costs for sulfamethoxazole-trimethoprim.*


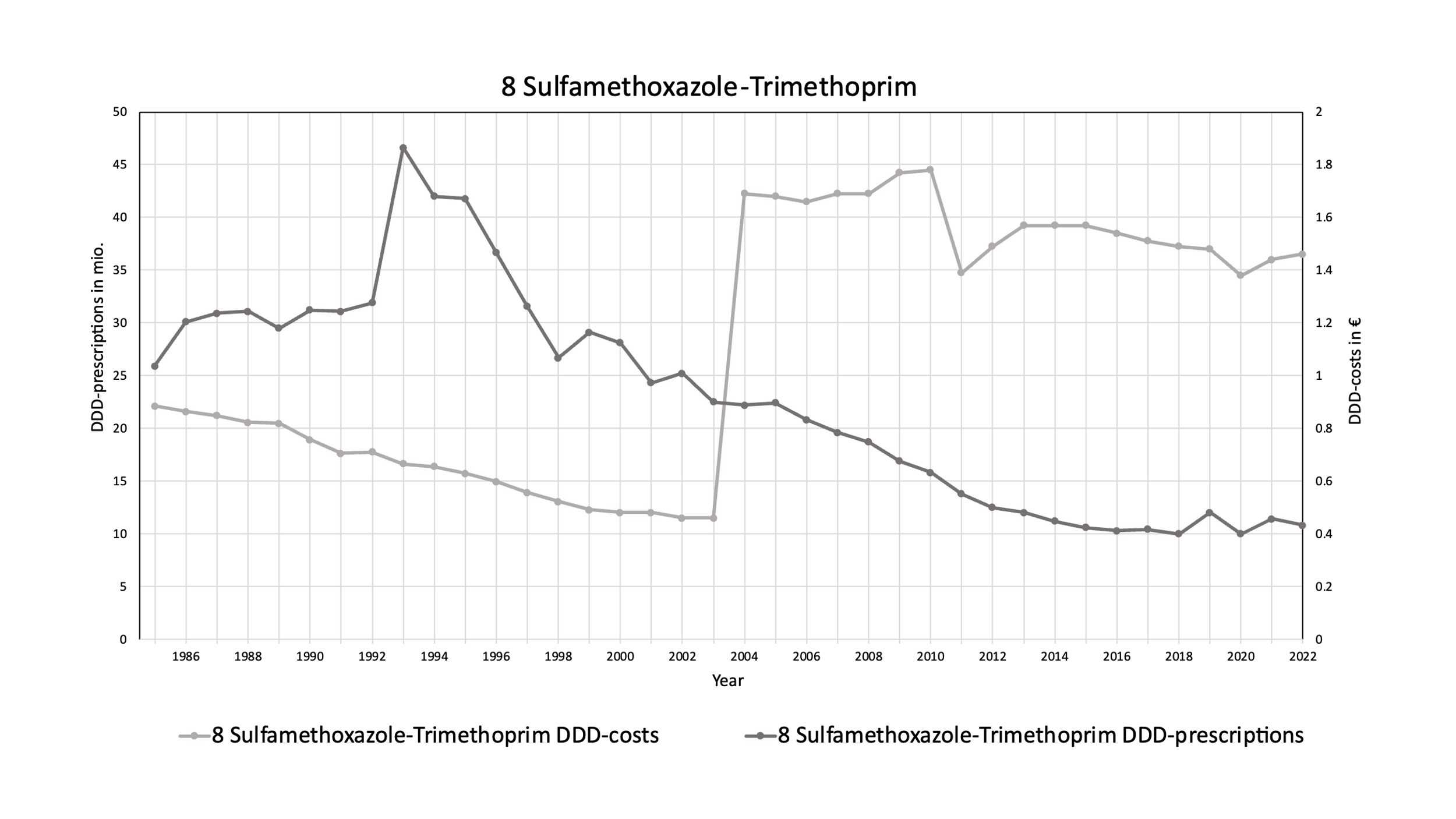


***Fig. S9:*** *Development of the DDD-prescriptions and DDD-costs for nitrofurantoin.*


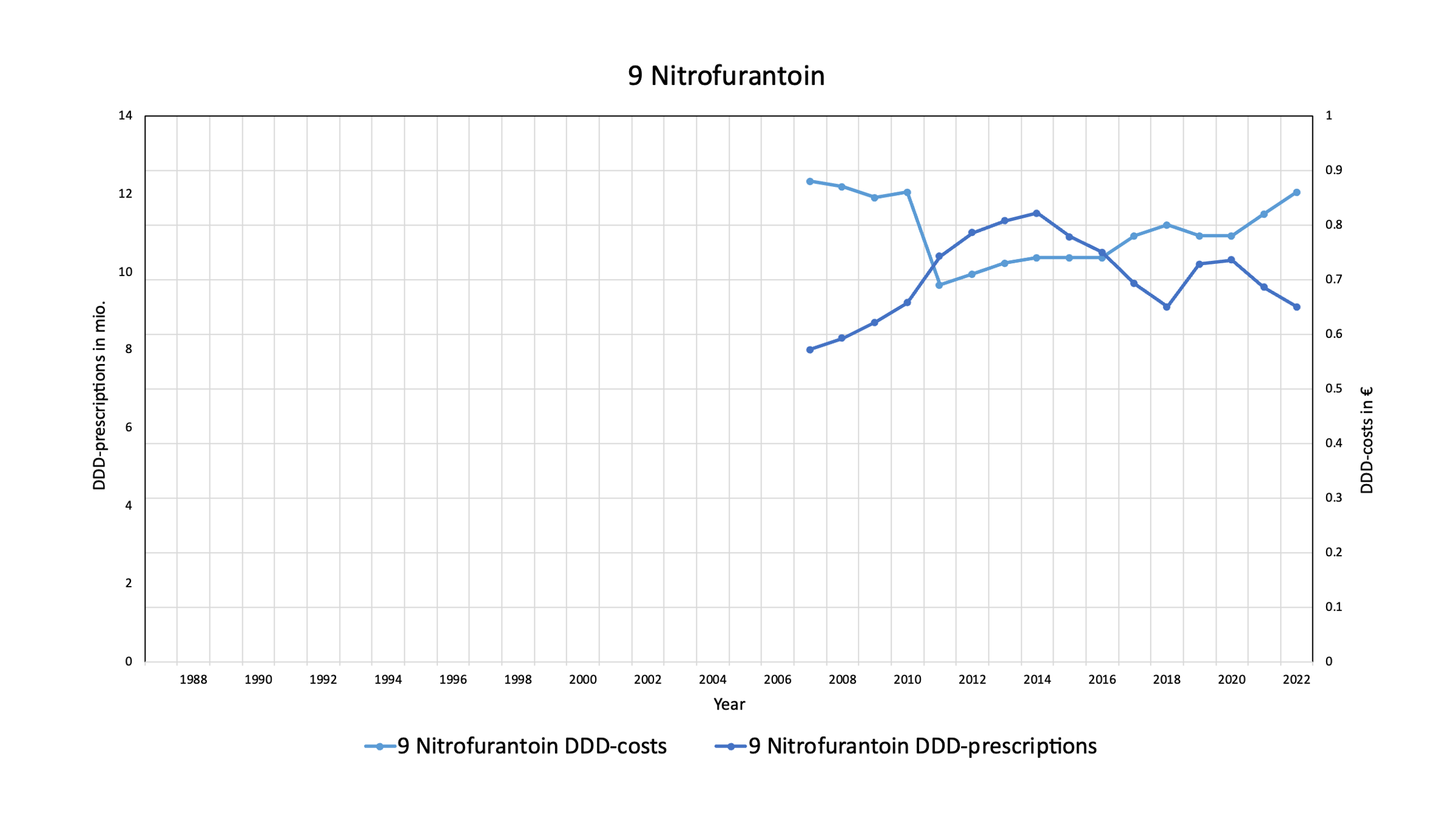


***Fig. S10:*** *Development of the DDD-prescriptions and DDD-costs for ciprofloxacin.*


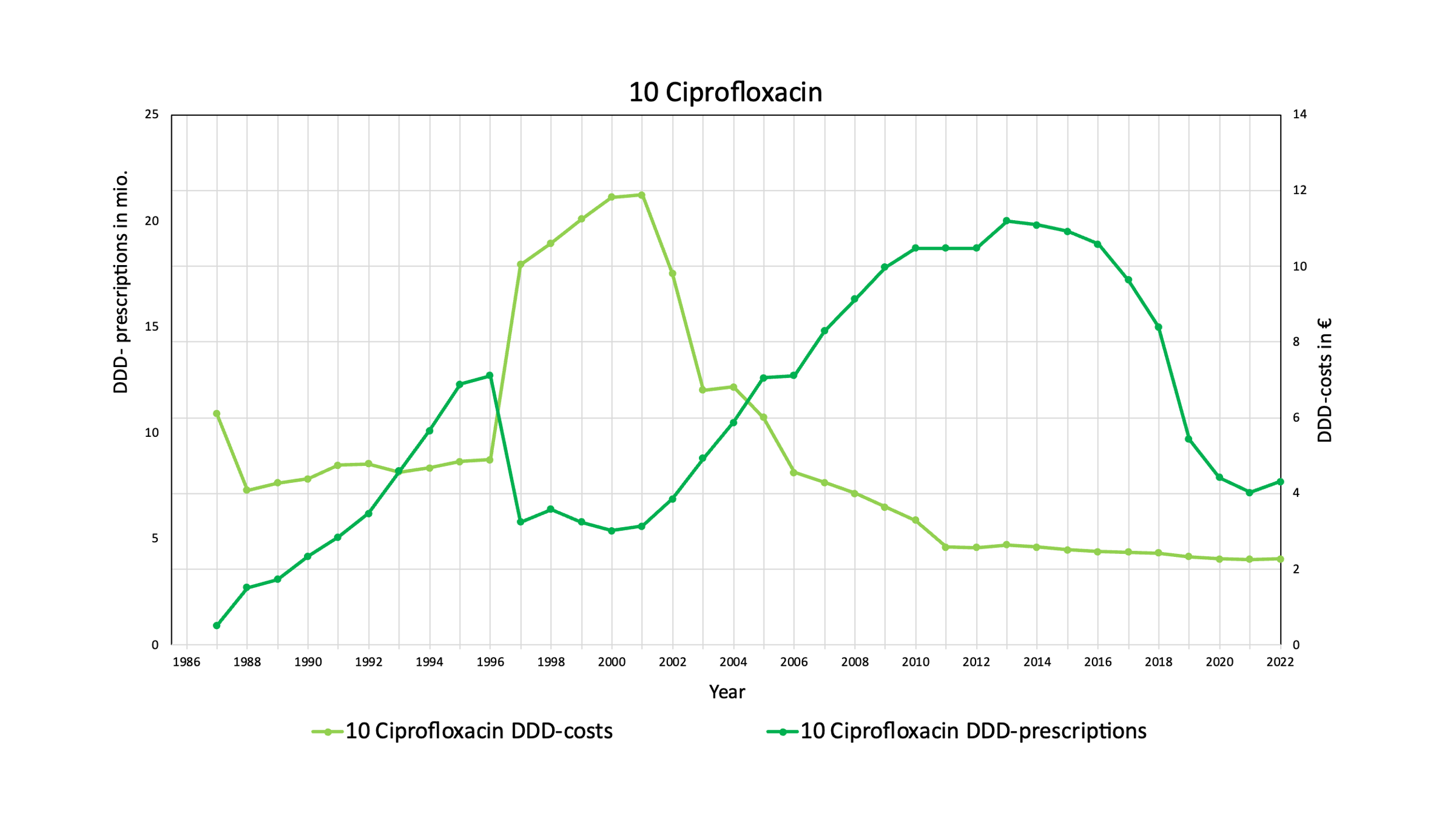


***Fig. S11:*** *Development of the DDD-prescriptions and DDD-costs for clarithromycin.*


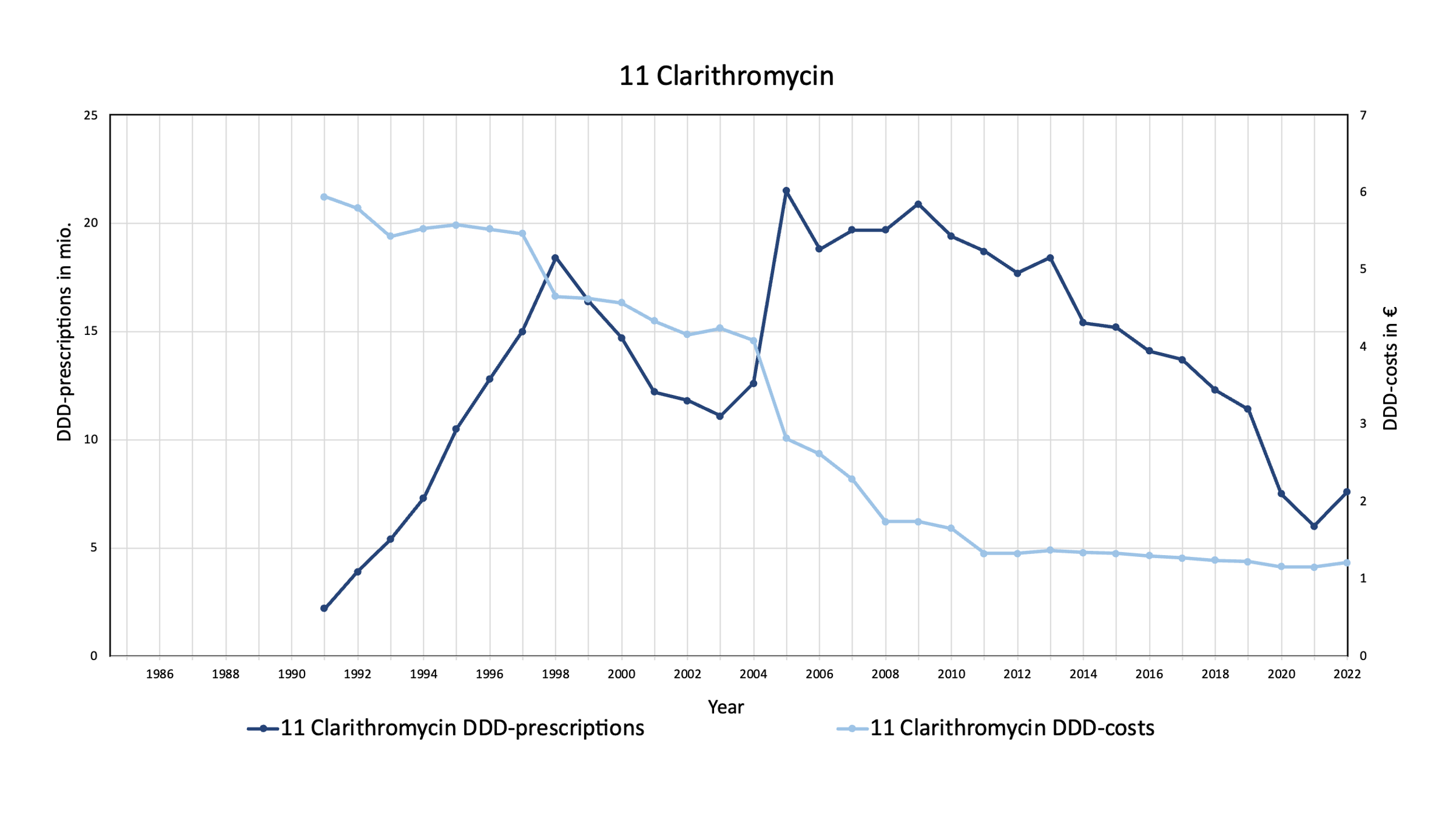


***Fig. S12:*** *Development of the DDD-prescriptions and DDD-costs for cefaclor.*


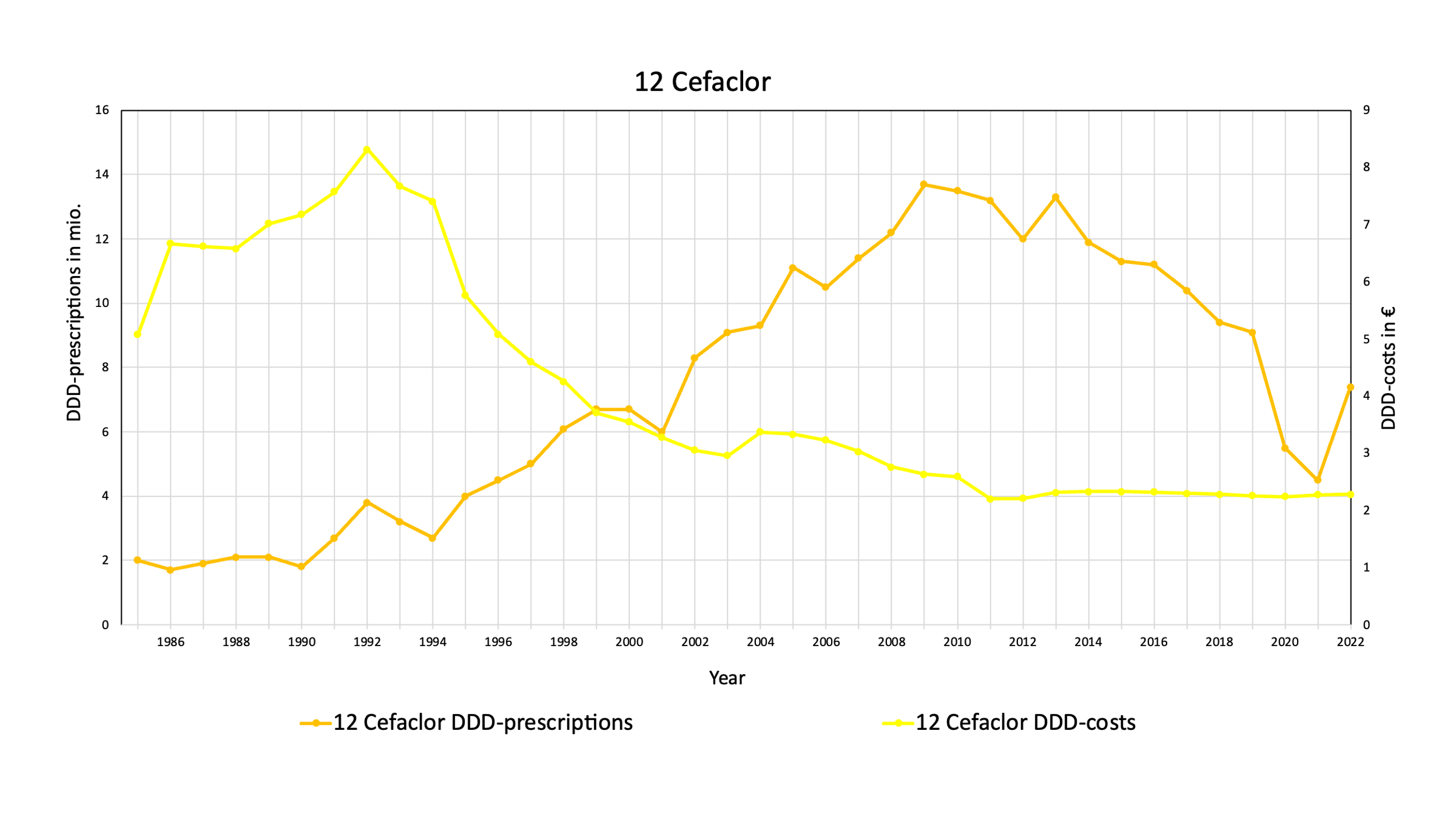


***Fig. S13:*** *Development of the DDD-prescriptions and DDD-costs for cefpodoxime.*


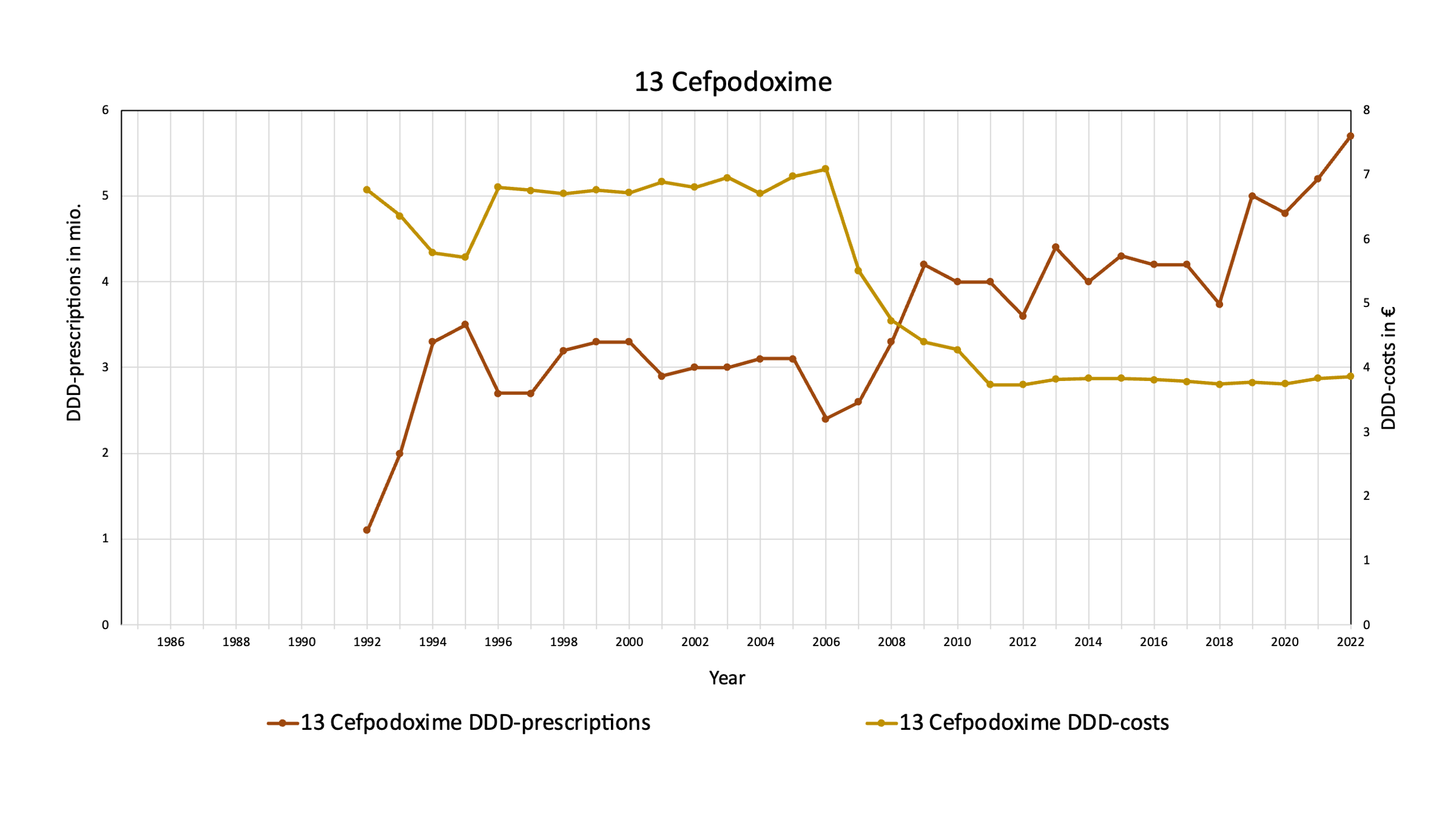


***Fig. S14:*** *Development of the DDD-prescriptions and DDD-costs for pivmecillinam.*


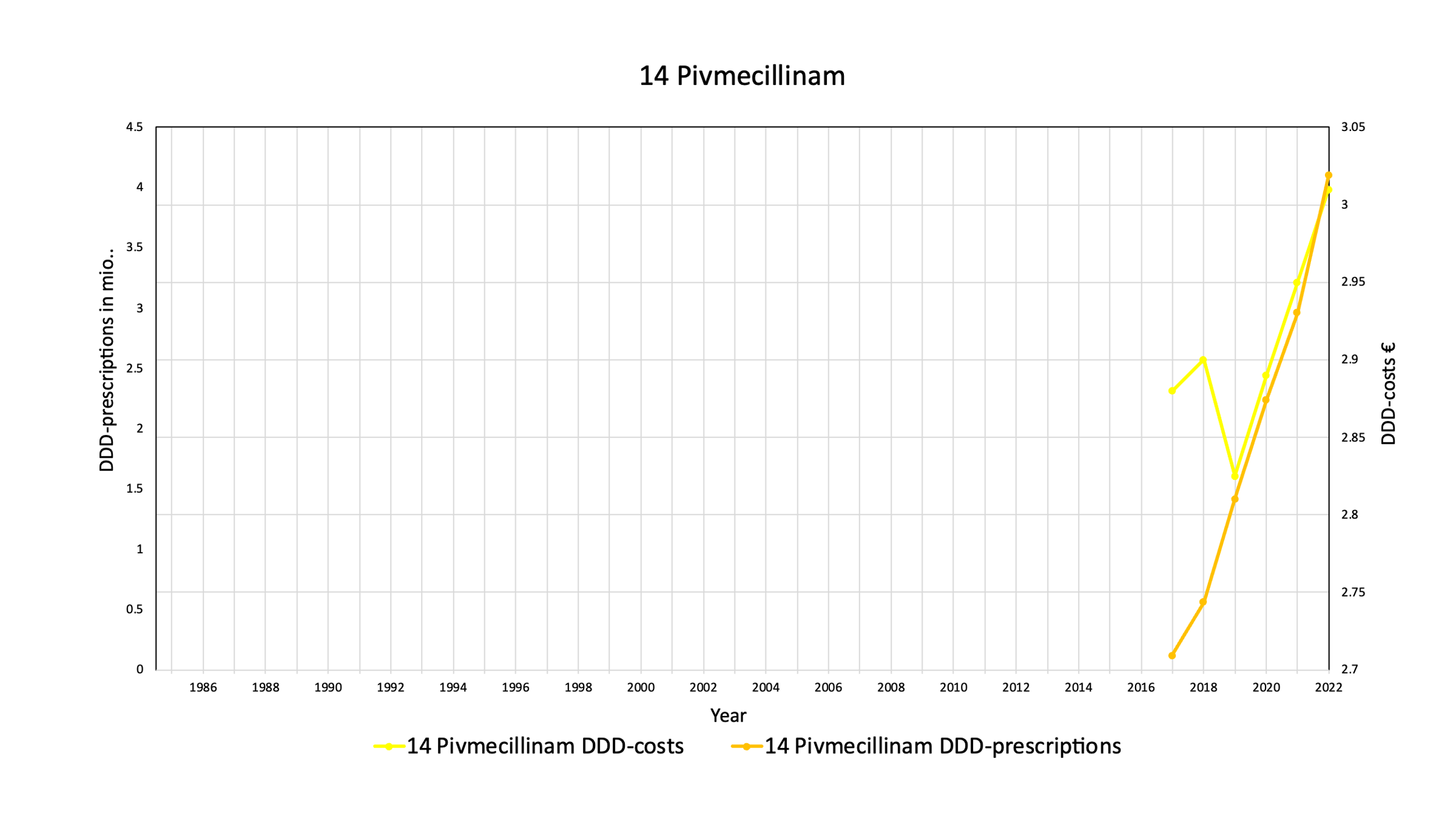


***Fig. S15:*** *Development of the DDD-prescriptions and DDD-costs for roxithromycin.*

*
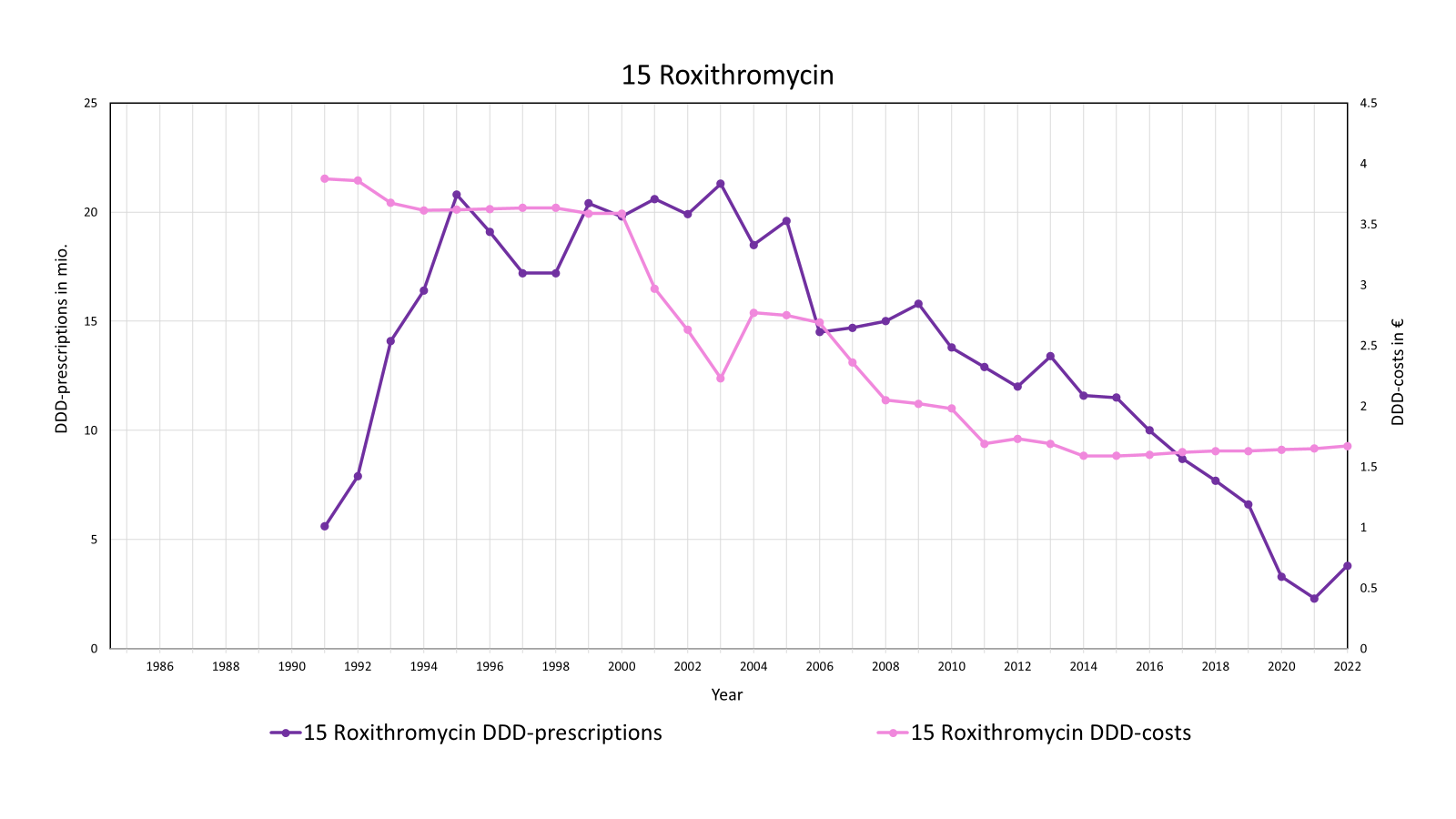
*
